# Effective use of legacy data in a genome-wide association studies improves the credibility of quantitative trait loci detection in rice

**DOI:** 10.1093/plphys/kiad018

**Published:** 2023-01-18

**Authors:** Mao Suganami, Soichi Kojima, Fanmiao Wang, Hideki Yoshida, Kotaro Miura, Yoichi Morinaka, Masao Watanabe, Tsukasa Matsuda, Eiji Yamamoto, Makoto Matsuoka

**Affiliations:** Faculty of Food and Agricultural Sciences, Institute of Fermentation Sciences, Fukushima University, Fukushima 960-1296, Japan; Graduate School of Agricultural Science, Tohoku University, Sendai 980-8572, Japan; Bioscience and Biotechnology Center, Nagoya University, Nagoya 464-8601, Japan; Faculty of Food and Agricultural Sciences, Institute of Fermentation Sciences, Fukushima University, Fukushima 960-1296, Japan; Faculty of Bioscience and Biotechnology, Fukui Prefectural University, Fukui 910-1195, Japan; Faculty of Bioscience and Biotechnology, Fukui Prefectural University, Fukui 910-1195, Japan; Graduate School of Life Sciences, Tohoku University, Sendai 980-8577, Japan; Faculty of Food and Agricultural Sciences, Institute of Fermentation Sciences, Fukushima University, Fukushima 960-1296, Japan; Graduate School of Agriculture, Meiji University, Kawasaki 214-8571, Japan; Faculty of Food and Agricultural Sciences, Institute of Fermentation Sciences, Fukushima University, Fukushima 960-1296, Japan

## Abstract

Genome-wide association studies (GWASs) are used to detect quantitative trait loci (QTL) using genomic and phenotypic data as inputs. While genomic data are obtained with high throughput and low cost, obtaining phenotypic data requires a large amount of effort and time. In past breeding programs, researchers and breeders have conducted a large number of phenotypic surveys and accumulated results as legacy data. In this study, we conducted a GWAS using phenotypic data of temperate *japonica* rice (*Oryza sativa*) varieties from a public database. The GWAS using the legacy data detected several known agriculturally important genes, indicating reliability of the legacy data for GWAS. By comparing the GWAS using legacy data (L-GWAS) and a GWAS using phenotypic data that we measured (M-GWAS), we detected reliable QTL for agronomically important traits. These results suggest that an L-GWAS is a strong alternative to replicate tests to confirm the reproducibility of QTL detected by an M-GWAS. In addition, because legacy data have often been accumulated for many traits, it is possible to evaluate the pleiotropic effect of the QTL identified for the specific trait that we focused on with respect to various other traits. This study demonstrates the effectiveness of using legacy data for GWASs and proposes the use of legacy data to accelerate genomic breeding.

## Introduction

Crop improvement through genomic breeding is essential to increase crop productivity and to feed the growing global population (Hickey et al., 2019). Identification and characterization of genes associated with agricultural traits not only offers an insight into the genetic basis of phenotypic variation but also contributes to efficient crop improvement. Genome-wide association studies (GWASs), which analyze the association between genome-wide nucleotide polymorphisms and phenotypic variations, have emerged as a powerful method in genetics (Myles et al., 2009; Hamblin et al., 2011; Huang and Han 2014; Lipka et al., 2015). GWASs require genomic data and phenotypic data as inputs. Genomic data can be obtained at high throughput with low cost due to rapid advances in sequencing technology (Huang et al., 2013; Nguyen et al., 2019). On the other hand, phenotyping of large populations usually requires a lot of effort, and, in many cases, replicate tests over several years are required to confirm reproducibility. Many landraces and cultivars have been characterized by researchers and breeders, and a large amount of phenotypic data have already been accumulated, referred to as “legacy data.” We hypothesized that if the legacy data could serve as phenotypic data for GWASs to detect useful quantitative trait loci (QTL) for agricultural production, it would greatly reduce the effort of phenotyping and accelerate genomic breeding.

To evaluate the validity of legacy data, we performed a GWAS on various rice phenotypic data retrieved from a public database provided by the NARO Genebank in Japan (https://www.gene.affrc.go.jp/distribution-plant_en.php). We performed the GWAS with 198 temperate *japonica* rice (*Oryza sativa* L.) varieties and found several known agriculturally important genes. We also conducted a GWAS using phenotypic data that we measured, referred to as a measured-GWAS (M-GWAS), and compared this with the results of the GWAS using legacy data, referred to as a legacy-GWAS (L-GWAS). Several genomic regions were detected in both the M-GWAS and L-GWAS, where previously unknown QTL are probably located. This demonstrated that the combination of an L-GWAS and M-GWAS is effective in detecting reliable QTL. In addition, we evaluated the pleiotropic effects of QTL by comparing GWASs for multiple traits using legacy data. Here, we show the availability of legacy data for accelerating basic research and practical genomic breeding.

## Results

### Population structure and phenotypic data for L-GWAS

**Figure 1:**
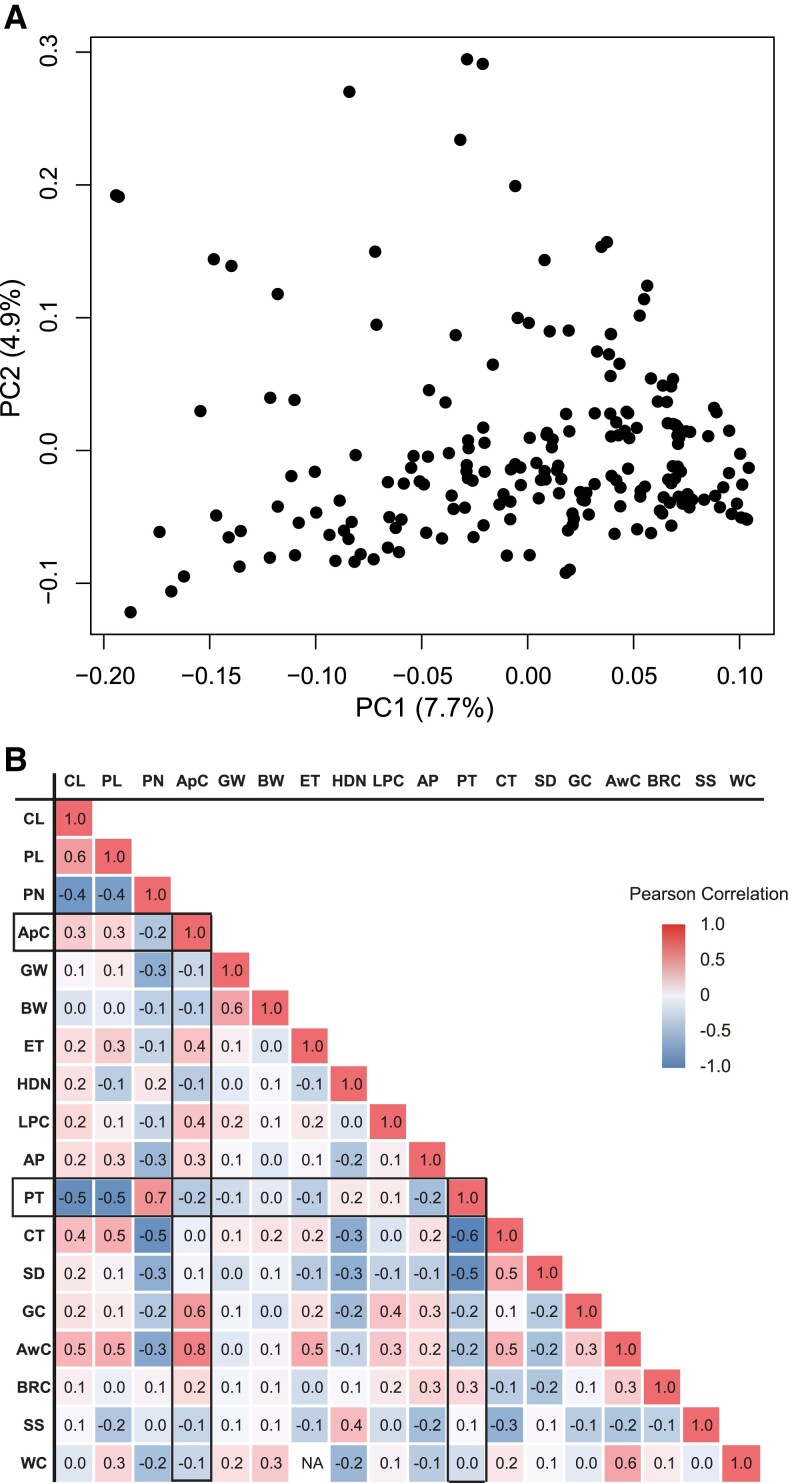
Genetic population structure of Japanese rice varieties used for L-GWAS and correlation matrix for 18 agronomic traits. A, PCA for the 198 varieties based on whole-genome data. PC1 and PC2 indicate the score of principal components 1 and 2, respectively. Values in parentheses indicate percentage of variance in the data explained by each principal component. B, Pearson correlation coefficients between phenotypic data from NARO Genebank. Blue and red indicate negative and positive correlations, respectively. The 18 traits are Culm Length (CL), Panicle Length (PL), Panicle Number (PN), Apiculus Color (ApC), Grain Width (GW), Brown rice Width (BW), Endosperm Type (ET), Heading Date at Niigata (HDN), Lemma and Palea Color (LPC), Awn Presence (AP), Plant Type (PT), Culm Thickness (CT), Spikelet Density (SD), Glume Color (GC), Awn Color (AwC), Brown Rice Color (BRC), Seed Shattering (SS), and amount of White Core (WC).

To perform GWASs efficiently, a genetically highly structured population is not desirable. A principal component analysis (PCA) was conducted to measure the population structure of 198 temperate *japonica* varieties used in the L-GWAS (Supplemental Table S1) and 172 temperate *japonica* varieties used in the M-GWAS (Supplemental Table S2). For both populations, the scores plot of the first two principal components showed a continuous distribution with no distinct subpopulation clusters, indicating that these two populations are not highly structured (Figure 1A and Supplemental Figure 1). A total of 33 traits were retrieved from NARO Genebank database (Table 1 and Supplemental Figure 2). Among these 33 traits, we focused on 18 traits (Figure 1B). As shown in Figure 1B, we found the following relationships between traits: Apiculus Color (ApC) was highly correlated with Glume Color (GC) and Awn Color (AwC) (*r* = 0.6 and 0.8, respectively), and moderately correlated with Lemma and Palea Color (LPC) (*r* = 0.4), but not with Brown Rice Color (BRC; *r* = 0.2). Plant Type (PT) was highly positively correlated with Panicle Number (PN; *r* = 0.7), while negatively correlated with Culm Length (CL), Panicle Length (PL), Culm Thickness (CT), and Spikelet Density (SD) (*r* = −0.5, −0.5, −0.6, and −0.5, respectively). This suggests that the PT, which is evaluated by the breeders’ intuition, is the result of the integration of these traits. In this case, by performing a GWAS on PT and the individual traits and comparing the loci controlling these traits, we were able to examine the relationship between PT and these specific traits in detail (see below).

**Table 1:** List of 33 traits and their evaluation methods used in this study. The original description can be found on the NARO Genebank website

No.	Traits	Rank or measurement unit
1	Culm Length (CL)	cm
2	Panicle Length (PL)	cm
3	Panicle Number (PN)	Number per plant
4	Apiculus Color (ApC)	1: Straw, 2: Tawny, 3: Brown, 4: Red brown, 5: Light red, 6: Red, 7: Light purple, 8: Purple, 9: Blackish purple
5	Grain Length (GL)	mm
6	Grain Width (GW)	mm
7	Brown rice Length (BL)	mm
8	Brown rice Width (BW)	mm
9	Endosperm Type (ET)	2: Non-glutinous, 8: Glutinous
10	Heading Date at Niigata (HDN)	Days from July 1
11	Integrated Heading Date corrected from 12 different places (IHD)	Days from July 1
12	Lemma and Palea Color (LPC)	1: Straw, 2: Yellow, 3: Gold, 4: Reddish yellow to orange, 5: Brown, 6: Reddish brown, 7: Purple, 8: Black, 9: Other
13	Awn Presence (AP)	0: Absent, 1: Extremely scarce, 2: Very scarce, 3: Scarce, 4: Slightly scarce, 5: Intermediate, 6: Slightly abundant, 7: Abundant, 8: Extremely abundant, 9: Complete
14	Awn Length (AL)	1: Very short, 3: Short, 5: Intermediate, 7: Long, 9: Very long
15	1,000 Grain Weight (TGW)	g
16	Plant Type (PT)	2: Super panicle weight type, 3: Panicle weight type, 4: Rather panicle weight type, 5: Intermediate type, 6: Rather panicle number type, 7: Panicle number type, 8: Super panicle number type
17	Culm Thickness (CT)	2: Very thin, 3: Thin, 4: Slightly thin, 5: Intermediate, 6: Slightly thick, 7: Thick, 8: Very thick
18	Flag Leaf Angle (FLA)	2: Erect, 3: Semi-erect, 4: Slightly semi-erect, 5: Intermediate, 6: Slightly descending, 7: Semi-descending, 8: Descending
19	Leaf Blade Color (LBC)	1: Yellow, 2: Yellowish blotched, 3: Light green, 4: Green, 5: Dark green, 6: Purple blotched, 7: Purple margin, 8: Purple, 9: Other
20	Spikelet Density (SD)	Number
21	Pubescence of Lemma and Palea (PLP)	0: None, 1: Rare, 2: Scarce, 3: Little, 4: Slightly little, 5: Intermediate, 6: Slightly abundant, 7: Abundant, 8: Very abundant, 9: Extremely abundant
22	Glume Color (GC)	1: Straw, 2: Gold, 3: Red, 4: Purple
23	Awn Color (AwC)	1: Straw, 2: Yellowish brown, 3: Brown, 4: Reddish brown, 5: Light red, 6: Red, 7: Light purple, 8: Purple, 9: Blackish purple
24	Brown Rice Color (BRC)	1: White, 2: Light brown, 3: Variegated brown, 4: Dark brown, 5: Light red, 6: Red, 7: Variegated purple, 8: Purple, 9: Dark Purple/black
25	Resistance to Leaf Blast (RLB)	1: Very high, 3: High, 4: Slightly high, 5: Intermediate, 6: Slightly low, 7: Low, 9: Very low
26	Lodging Tolerance (LT)	1: Very high, 3: High, 4: Slightly high, 5: Intermediate, 6: Slightly low, 7: Low, 9: Very low
27	Pre-Harvest Sprouting (PHS)	1: Very high, 3: High, 4: Slightly high, 5: Intermediate, 6: Slightly low, 7: Low, 9: Very low
28	Resistance to Sheath Blight (RSB)	1: Very high, 3: High, 4: Slightly high, 5: Intermediate, 6: Slightly low, 7: Low, 9: Very low
29	Seed Shattering (SS)	2: Very hard, 3: Hard, 4: Slightly hard, 5: Intermediate, 6: Slightly easy, 7: Easy, 8: Very easy
30	Grain Appearance (GA)	1: Extremely bad, 2: Very bad, 3: Bad, 4: Slightly bad, 5: Intermediate, 6: Slightly good, 7: Good, 8: Very good, 9: Excellent
31	Grain Luster (Glu)	2: Very low, 3: Low, 4: Slightly low, 5: Intermediate, 6: Slightly high, 7: High, 8: Very high
32	Amount of White Belly (WB)	2: Very low, 3: Low, 4: Slightly low, 5: Intermediate, 6: Slightly high, 7: High, 8: Very high
33	Amount of White Core (WC)	2: Extremely few, 3: Very few, 4: Few, 5: Intermediate, 6: Some, 7: Many, 8: Very many

The distributions of the 33 traits in the legacy data were examined in histograms (Supplemental Figure 3). Among these, 13 traits that tended to be extremely unidirectional (highlighted in blue in Supplemental Figure 3) were analyzed as binary traits, and the other 20 traits (highlighted in green) were analyzed as quantitative traits. Fifteen of the traits are specifically discussed in this paper, while the results for the other traits are shown in Supplemental Figure 4. We confirmed all quantile–quantile (Q–Q) plots and found no statistical problems in the GWASs (Supplemental Figures 5 and 6).

### Validity of L-GWAS

**Figure 2:**
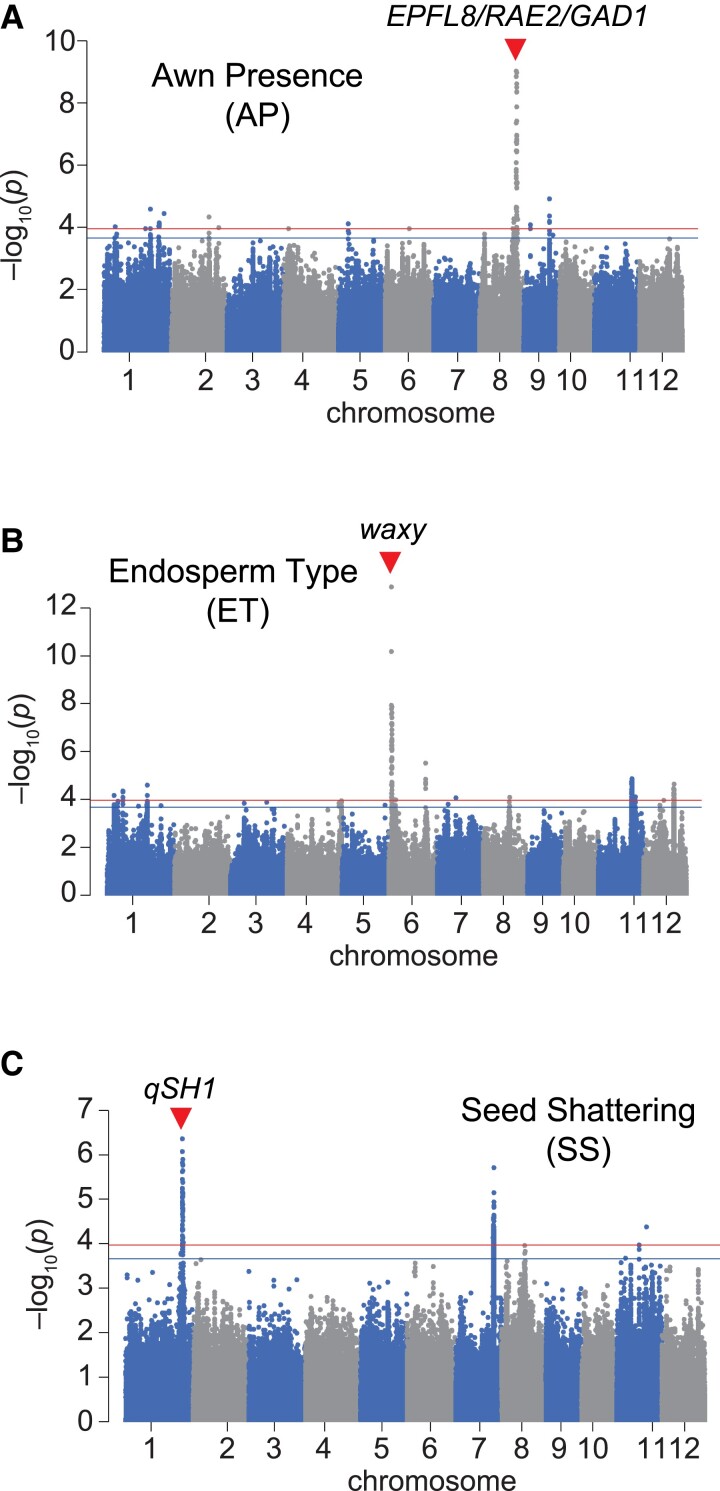
L-GWAS of three agronomic traits for which genes that have a major effect on each trait have been identified. Manhattan plot of L-GWAS of Awn Presence (AP) (A), Endosperm Type (ET) (B), and Seed Shattering (SS) (C). Red arrowheads indicate the position of the gene previously reported to control each trait, that is *EPFL8/RAE2/GAD1* for awn formation, *waxy* for endosperm starch synthesis, and *qSH1* for SS. Genome-wide significant threshold is indicated by horizontal lines (red: 0.1/*M*_eff_, blue: 0.2/*M*_eff_).

First, we focused on three traits, Awn Presence (AP), Endosperm Type (ET), and Seed Shattering (SS), which we considered to be suitable for evaluating whether the L-GWAS is effective because genes that have a major effect on each trait have been identified. In the GWAS for AP, a strong peak was detected on chromosome (Chr.) 8 (Figure 2A); within the linkage disequilibrium block of this peak, *Epidermal Patterning Factor-Like protein 8 (EPFL8)/Regulator of Awn Elongation 2 (RAE2)/Grain number*, *grain length and Awn Development 1 (GAD1)* is located (Yano et al., 2016; Bessho-Uehara et al., 2016; Jin et al., 2016). A strong peak was also detected at the same position on Chr. 8 in the GWAS for Awn Length (AL) (Supplemental Figure 4). In the GWAS for ET, a strong peak was detected on Chr. 6 (Figure 2B), where *waxy* is located (Inukai et al., 2000). In the GWAS of SS, a strong peak was detected on Chr. 1 (Figure 2C), where *QTL of seed shattering in chromosome 1 (qSH1)* is located (Konishi et al., 2006). These results demonstrate that the L-GWAS works effectively to easily identify QTL controlling agronomic traits.

### Combination of L-GWAS and M-GWAS for finding QTL

**Figure 3:**
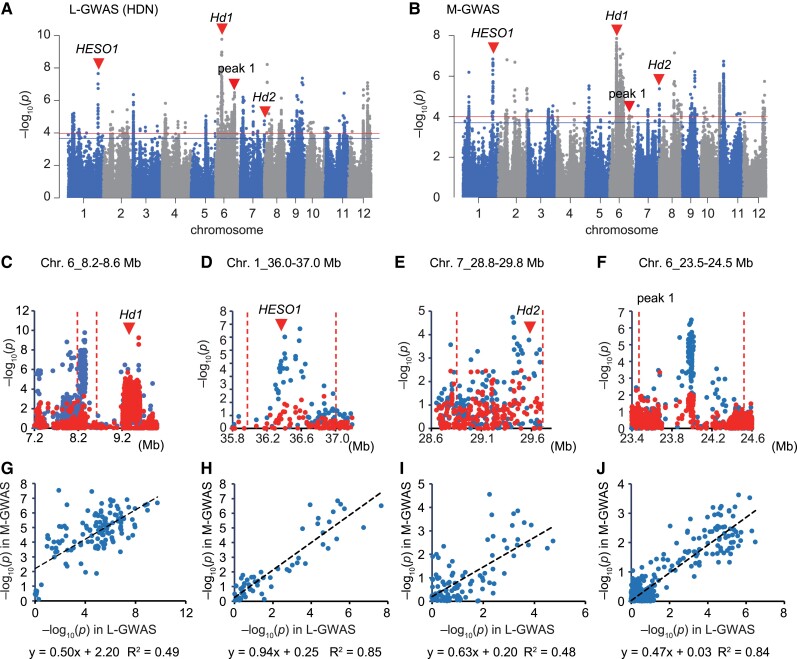
Comparison between L- and M-GWASs for heading date. A–B, Manhattan plot of Heading Date at Niigata (HDN) using legacy data (A) and data that we measured at Nagoya (B). Red arrowheads indicate peaks observed in both L- and M-GWASs. Three known heading genes, *Hd1*, *HESO1*, and *Hd2*, colocalize in these peak regions. Genome-wide significant threshold is indicated by horizontal lines (red: 0.1/*M*_eff_, blue: 0.2/*M*_eff_). C–F, Local Manhattan plot of L-GWAS surrounding the peak regions indicted by red arrowheads in the panels (A, B). Plots show the results of L-GWAS performed without (blue) or with (red) the polymorphism with the highest signal in M-GWAS as fixed effect. Red arrowheads indicate the position of heading genes, that is *Hd1* (C), *HESO1* (D), and *Hd2* (E), while there is no known heading gene in panel (F). Dashed lines indicate the candidate region for the peak. G–J, Correlation of the −log_10_(*P*) value of SNPs between L- and M-GWASs within the peak regions of *Hd1*, *HESO1*, *Hd2*, and peak 1.

The above three traits are the easiest cases for GWASs because these traits are strongly affected by a single major QTL. Therefore, we next focused on GWASs for traits controlled by multiple QTL and compared the L-GWAS with the GWAS using the phenotypic data we measured (M-GWAS). For this purpose, we first focused on comparing results from the L-GWAS for Heading Date at Niigata (HDN) and those from the M-GWAS for the Heading Date measured at Nagoya. There were several peaks in the Manhattan plot of the L-GWAS; some of these overlapped with known heading genes, such as *HEN1 suppressor 1 (HESO1)*, *Heading date 1 (Hd1)*, and *Heading date 2 (Hd2)* (Figure 3A; Yano et al., 2016), and these peaks were also detected in the M-GWAS (Figure 3B). For *Hd1* (Figure 3C), we previously reported that a strong indirect association occurs in the near region due to many different haplotypes of *Hd1*, resulting in a shift in peak position when we used Japanese *japonica* varieties (Yano et al., 2016; Tibbs Cortes et al., 2021).

To confirm the consistency of the L- and M-GWASs, we applied two methods. First, we performed an L-GWAS including the polymorphism with the highest signal in the M-GWAS as a fixed effect to determine whether the peak of interest disappeared (Method 1). Application of Method 1 to the *Hd1*, *HESO1*, and *Hd2* peaks resulted in disappearance of these peaks (Figure 3, C–E). For Method 2, we examined the correlation of −log_10_(*P*) of single-nucleotide polymorphisms (SNPs) between the L-GWAS and M-GWAS within the peak regions (Figure 3, G–I). In the case of the peak region on Chr. 1_36.0–37.0 Mb, where *HESO1* is located (Figure 3D), there were 60 polymorphisms shared between the L- and M-GWASs. The correlation of ­–log_10_(*P*) of these polymorphisms was very high (coefficient of determination; *R*^2^ = 0.85) between the L- and M-GWASs (Figure 3H). In a similar fashion, we tested the equivalence of the L- and M-GWAS peaks in the *Hd1* and *Hd2* regions and found a high correlation between these peaks (Figure 3, C, E, G, and I). We also applied these methods for peak 1, which was located at around 24 Mb on Chr. 6 in both the L- and M-GWASs (Figure 3, A and B). Although no known flowering genes have been reported in this region (RiceNavi; Wei et al., 2021), the peak disappeared using Method 1 (Figure 3F) and a high correlation was confirmed using Method 2 (*R*^2^ = 0.84; Figure 3J). These results demonstrate that these two peaks are identical and, thus, the presence of a previously unknown heading QTL in this region is indicated. Hereafter, we used these two methods as an “identity test” for comparison of peaks from L- and M-GWASs.

**Figure 4:**
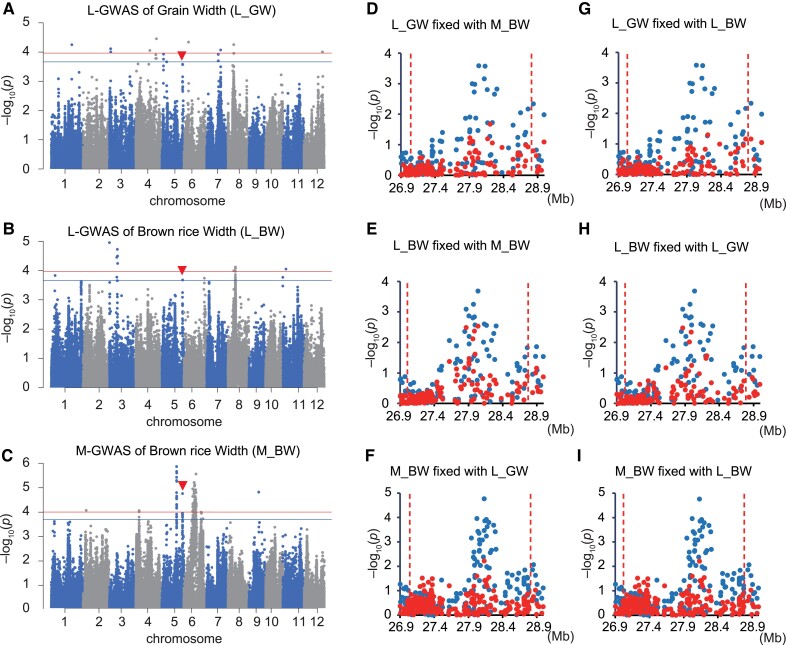
Comparison between L- and M-GWAS for seed width. A–C, Manhattan plot of Grain Width (GW) and Brown rice Width (BW) using legacy data (L_GW; A, L_BW; B) and of BW using data that we measured (M_BW; C). Red arrowheads indicate a peak observed in both L- and M-GWASs. Genome-wide significant threshold is indicated by horizontal lines (red: 0.1/*M*_eff_, blue: 0.2/*M*_eff_). D–I, Local Manhattan plot of L_GW (D, G), L_BW (E, H), and M_BW (F, I) surrounding the peak regions indicted by red arrowheads in the panels (A–C). Blue circles are the results of GWAS performed without fixed effect. Red circles are the results of GWAS performed with the polymorphism with the highest signal in M_BW (D, E), L_GW (F, H), and L_BW (G, I) as fixed effect.

For the above L-GWAS on Heading Date, we used the NARO phenotypic data from only a single location (i.e. Niigata). Data of Heading Date measured at different locations are available in legacy data of NARO Genebank and using these data would improve the statistical accuracy of the GWAS by increasing repeatability and decreasing missing values. However, there is also concern that mixing data from different locations could reduce the accuracy of the GWAS, because the influence of a QTL could differ between different locations. With these considerations in mind, we conducted an L-GWAS of the Integrated Heading Date (IHD) using all data recorded at all different locations (Supplemental Figure 7A). All four peaks discussed above (*Hd1*, *HESO1*, *Hd2*, and peak1) were also detected in this L-GWAS (Supplemental Figure 7, B–I). In addition to the four peaks, we found an additional peak (peak 2 hereinafter) on Chr. 6_3.4–3.7 Mb (Supplemental Figure 7A). The identity test showed peak disappearance using Method 1 (Supplemental Figure 7J) and a high correlation using Method 2 (*R*^2^ = 0.56; Supplemental Figure 7K), indicating that the QTL detected in the L- and M-GWASs are identical. To the best of our knowledge, there is no gene involved in heading date in this region, indicating that the peak includes a previously unknown QTL for heading date. These observations suggest that performing a GWAS with multiple datasets generated from legacy data is a realistic and effective way to ensure reproducibility and efficiently identify QTL.By using the identity test, we also attempted to detect QTL for Grain Width (GW) using an L-GWAS (L_GW; Figure 4A) and Brown rice Width (BW) using both an L- and an M-GWAS (L_BW and M_BW; Figure 4, B and C). We found a major peak on Chr. 5_27.0–28.8 Mb (Figure 4, A–C), with a high correlation according to Method 2 (*R*^2^ = 0.56, 0.64, and 0.40; Supplemental Figure 8, A–C). Using Method 1, we tested all combinations of the three GWASs (L_GW, L_BW, and M_BW) using the highest signal polymorphism in each GWAS. When L_GW and M_BW were compared, peak disappearance was observed in both cases (Figure 4, D and F), indicating that these two peaks are identical. Because we could not find genes whose relationship with the traits has been validated, we concluded that there is a previously unknown QTL in this region. When we performed a GWAS of L_GW and M_BW, including the highest signal in L_BW as a fixed effect, the peaks disappeared (Figure 4, G and I). On the other hand, when we performed a GWAS of L_BW, including the highest signal in L_GW and M_BW, the peak on Chr. 5_27.0–28.8 Mb was decreased but did not disappear completely (Figure 4, E and H). These results suggest that the L_BW peak is shared with that of L_GW and M_BW, whereas there may also be another QTL in the same region for L_BW. These results indicate that even if peaks from independent GWAS results appear to be identical, the hypothesis must be examined carefully using the identity test.

**Figure 5:**
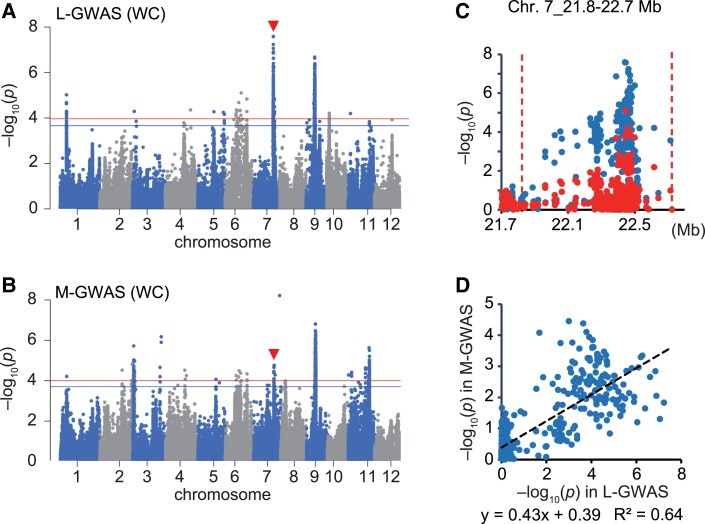
Comparison between L- and M-GWASs for amount of white core. A, B, Manhattan plot of amount of White Core (WC) by using legacy data (A) and using data that we measured (B). Red arrowheads indicate a peak observed in both L- and M-GWASs. Genome-wide significant threshold is indicated by horizontal lines (red: 0.1/*M*_eff_, blue: 0.2/*M*_eff_). C, Local Manhattan plot of L-GWAS surrounding the peak regions indicted by red arrowheads in the panels (A, B). Plots show the results of L-GWAS performed without (blue) or with (red) the polymorphism with the highest signal in M-GWAS as a fixed effect. D, Correlation of the −log_10_(*P*) value of SNPs between L- and M-GWASs within the peak region.

In the same way, we compared GWASs on the amount of White Core (WC) using the legacy data and the data that we measured (Figure 5, A and B). Because the GWAS platform used for binary traits in this study was not available for Method 1 (i.e. there was not an option to add arbitrary fixed effects), we performed GWASs for WC with the platform for quantitative traits (Figure 5, A and B). We found a major peak on Chr. 7_21.8–22.7 Mb, and the identity test showed peak disappearance using Method 1 (Figure 5C) and a high correlation using Method 2 (*R*^2^ = 0.64; Figure 5D). Also, in the GWAS for binary traits, the peak was detected in the same region in both the L-GWAS and M-GWAS (Supplemental Figure 9, A and B) and showed a high correlation (*R*^2^ = 0.56; Supplemental Figure 9, C and D). Because we could not find any genes in this region that have been verified to be related to the trait, we concluded that there is a previously unknown QTL for WC in this region.

These case studies show that the combination of L- and M-GWASs is an easy and available method to find QTL for agronomically important traits. In particular, WC is known to be greatly affected by environment, such as temperature during the ripening period (Sreenivasulu et al., 2015), and is considered to be a difficult trait for QTL analysis. However, we can ensure the reliability of the detected QTL from the data we collected by examining the reproducibility of the results from the L-GWAS, which uses completely independent phenotypic data (see “Discussion”).

### Evaluation of pleiotropic impacts of QTL by L-GWAS

**Figure 6:**
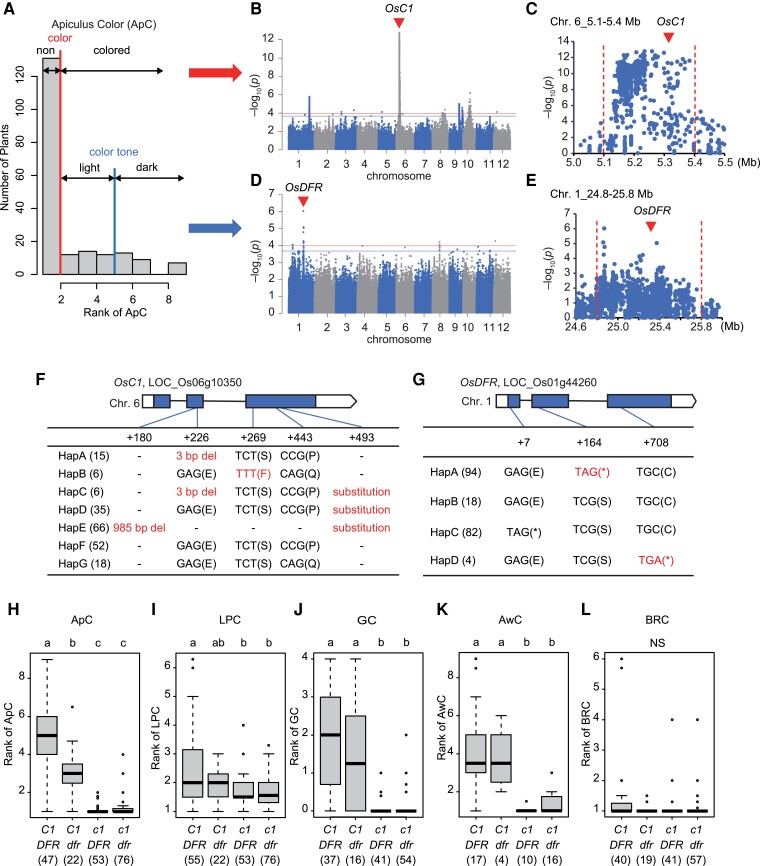
Evaluation of epistatic and pleiotropic effects on five color-related traits. A, Histogram of Apiculus Color (ApC), which was rated by NARO as 1–9 based on color differences. Red and blue line separates colored/non-colored and light/dark color, respectively. B, D, Manhattan plot based on colored/non-colored (B), or light/dark color (D). Arrowheads indicate the top peak colocalized with *OsC1* and *OsDFR*. Genome-wide significant threshold is indicated by horizontal lines (red: 0.1/*M*_eff_, blue: 0.2/*M*_eff_). C, E, Local Manhattan plot surrounding the top peaks in panels (B) and (D). F, G, Exon-intron structure of *OsC1* and *OsDFR* and DNA polymorphisms of these genes found in the legacy panel of 198 lines. Of these polymorphisms, mutations shown in red are thought to disrupt gene function (Sun et al., 2018; Zheng et al., 2019; Meng et al., 2021). The number of rice varieties in the 198 panel is shown in parentheses. H–L, Boxplots for five color-related traits: ApC (H), Lemma and Palea Color (LPC) (I), Glume Color (GC) (J), Awn Color (AwC) (K), and Brown Rice Color (BRC) (L). In the box plot, the box height shows the 25th and 75th quantiles, the whiskers are min–max values, the horizontal line is the median, black dots are the outliers. Upper- and lower-case letters indicate functional and null of these genes. Values in parentheses indicate sample numbers. Different letters above each box indicate significant differences among the genotypes as determined by Tukey–Kramer HSD post hoc tests (*P* < 0.05). NS means differences among the genotypes are not statistically significant by ANOVA (*P* > 0.05).

As another example of the efficient application of legacy data, we evaluated the pleiotropic impact of QTL using legacy data. As discussed above (Figure 1B), ApC correlated with the other color-related traits, LPC, GC, and AwC, but not with BRC. Here, a GWAS of ApC based on the color variation scored in the NARO phenotypic data (scores 1–9; Table 1) was performed as follows. First, a binary-GWAS of color (non-colored [scores 1] and colored [scores 2–9]) detected a strong peak on Chr.6_5.1–5.4 Mb, which contains R2R3-MYB gene (*OsC1*) (Figure 6, B and C). Second, a binary-GWAS of color tone (light-color [scores 2–4] and dark-color [scores 5–9]) detected a peak on Chr.1_24.8–25.8 Mb containing the dihydroflavonol 4 reductase gene (*OsDFR*) (Figure 6, D and E). There are seven haplotypes of *OsC1* in the GWAS panel, where Hap A-E corresponds to the null allele (Sun et al., 2018; Zheng et al., 2019; Meng et al., 2021) (Figure 6F). In *OsDFR*, there are four haplotypes and Hap A and D are null (Sun et al., 2018; Zheng et al., 2019) (Figure 6G). Based on this information, we examined the epistatic and pleiotropic effects of *OsC1* and *OsDFR* on ApC (Figure 6H) and the other four color-related traits (Figure 6, I–L). With a few exceptions, *OsC1* null lines (*osc1*) had a non-colored ApC regardless of the haplotype of *OsDFR*, while the functional *OsC1* lines had a colored ApC (Figure 6H). For the exceptional lines that showed discrepancy between *OsC1* haplotype and ApC phenotype, we rechecked their ApC ourselves. Two of them that carried the *OsC1* haplotype and were recorded as non-colored we found to be colored, while three of them that carried *osc1* and were recorded as colored we found to be non-colored (Supplemental Figure 10). *OsC1*/*OsDFR* lines showed a dark-color (scores 5–9) for ApC, while *OsC1*/*osdfr* showed a light-color (scores 2–4) (Figure 6H). These results are consistent with the GWAS results (Figure 6, A–E), suggesting that *OsC1* determines whether the apiculus is colored or non-colored whereas *OsDFR* regulates the degree of ApC (i.e. color tone). Previous studies have shown that *OsC1* acts as a switching gene for regulation of anthocyanin synthesis and activation of the expression of *OsDFR* and other related genes, and *OsDFR* is involved in the branching of anthocyanin synthesis pathways (Sun et al., 2018). The results for LPC, GC, and AwC demonstrate that functional *OsC1* is essential for colorations of those organs (Figure 6, I–K). On the other hand, among the organ color phenotypes, BRC was not associated with haplotypes of *OsC1* and/or *OsDFR* (Figure 6L), indicating that BRC is not controlled by these genes.

**Figure 7:**
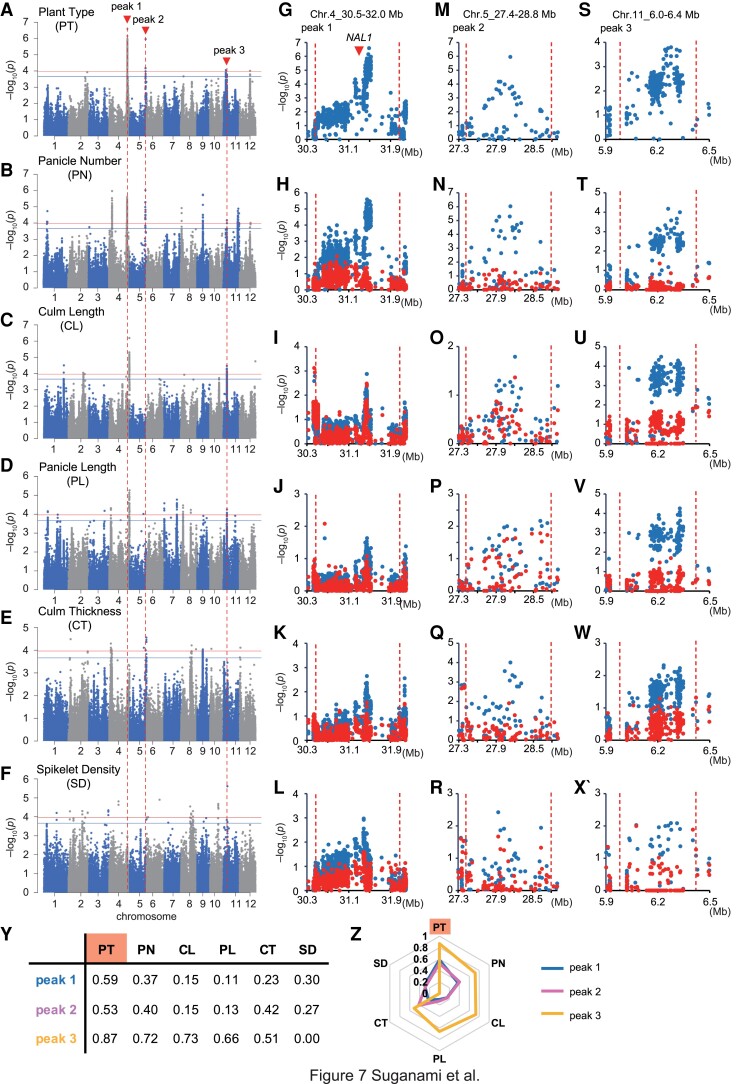
Breakdown of plant type into five measurable traits. A–F, Manhattan plot of Plant Type (PT) (A), Panicle Number (PN) (B), Culm Length (CL) (C), Panicle Length (PL) (D), Culm Thickness (CT) (E), and Spikelet Density (SD) (F). Arrowheads indicate candidate peaks common to PT and other traits. Genome-wide significant threshold is indicated by horizontal lines (red: 0.1/ *M*_eff_, blue: 0.2/ *M*_eff_). G–X, Local Manhattan plot of PT and five traits surrounding peak 1 (G–L), peak 2 (M–R), and peak 3 (S–X). Plots show the results of GWAS performed without (blue) or with (red) the polymorphism with the highest signal in PT as fixed effect. Red arrowheads indicate the position of *NAL1.* Y, Table and (Z) Rader chart of effect size of peaks 1–3 on PT and five traits.

PT is a trait evaluated by breeders’ intuition and has been considered to encompass several morphological traits. According to the correlation matrix (Figure 1B), we conducted a GWAS for five traits related to PT, namely PN, CL, PL, CT, and SD (Figure 7, A–F). Based on the GWAS of PT, we focused on the top three peaks (Figure 7A), and examined whether the five traits had the equivalent peaks by Method 1 of the identity test using the polymorphism with the highest signal of PT as a fixed effect. Peak 1 on Chr. 4_30.5–32.0 Mb contained *NALLOW LEAF 1* (*NAL1*) (Figure 7G), which controls panicle size, flag leaf width, and PN, as previously reported (Fujita et al., 2013; Yano et al., 2016). From our analysis, PN showed the strong peak in the peak 1 region and the peak disappeared by Method 1 (Figure 7H). The −log_10_(*P*) values of CT and SD were relatively low, but the peak disappeared by Method 1 (Figure 7, K and L). On the other hand, CL and PL did not show the peak disappearance (Figure 7, I and J). Thus, peak 1, including *NAL1*, is involved in determining PT, through PN predominantly and CT and SD moderately. For peak 2 on Chr. 5_27.4–28.8 Mb (Figure 7M), PN showed a strong peak and the peak disappeared by Method 1 (Figure 7N). CT also showed peak disappearance (Figure 7Q), but CL, PL, and SD did not show the peak disappearance (Figure 7, O, P, and R). Chigira et al. (2020) reported the presence of a pleiotropic QTL in the same region, which affects multiple traits including PN and lodging resistance. We performed an M-GWAS using the PN data from Chigira et al. (2020) (Supplemental Figure 11A) and compared it to our L-GWAS of PN (Figure 7B). Peak 2 disappeared in the L-GWAS when Method 1 was applied using the polymorphism of the highest signal in the M-GWAS (Supplemental Figure 11B), suggesting that peak 2 is identical to the QTL reported in Chigira et al. (2020). For peak 3 on Chr. 11_6.0–6.4 Mb (Figure 7S), PN, CL, and PL showed a strong peak and the peak disappeared by Method 1 (Figure 7, T–V). The −log_10_(*P*) values of CT were relatively low, but the peak disappeared by Method 1 (Figure 7W). On the other hand, SD did not show the peak disappearance (Figure 7X). Consequently, unlike peaks 1 and 2, peak 3 is involved in PT via PN and length-related traits, namely, CL and PL. To calculate the effect size of peaks 1–3 on each trait, we performed a GWAS using the standardized phenotypic data (Figure 7, Y and Z). The results indicated that all peaks affect PT, and peak 3 is the QTL that has the greatest impact on PT. Peaks 1 and 2 affect PN predominantly and CT and SD moderately, whereas peak 3 is a pleiotropic QTL affecting PN, CL, PL and CT (Figure 7Z). These results are consistent with the results of the identity test described above.

## Discussion

Using rice phenotypic data from the NARO Genebank as an example, this study shows that legacy data are very useful for GWASs. The advantages of using legacy data revealed by this study are as follows. First, the process of acquiring trait data, which requires a lot of time and effort, can be greatly reduced by allowing legacy data to be used in place of newly acquired data, as like a kind of blinded experiment. Also, by comparing the legacy data and our own acquired data, simple mistakes (e.g. color observation error in legacy data; Supplemental Figure 10) can be eliminated. In addition, because in many cases the populations that researchers have analyzed and populations in legacy data are different in terms of contents (varieties), the possibility of statistical errors will be greatly reduced compared to that in GWASs using a single population. In this context, GWASs using two independent populations with independently acquired trait data would be useful for reliable QTL detection. In this study, we succeeded in detecting previously unknown QTL for three agronomic traits by this method: Heading Date (Figure 3), Seed Width (Figure 4), and WC (Figure 5). In particular, a reproducible QTL was detected even for WC (Figure 5), which is greatly affected by environmental factors, suggesting that the use of legacy data is very effective for reliable QTL detection. Furthermore, legacy data, like the NARO Genebank used in this study, often contain a comprehensive range of phenotypic data. Thus, it is possible to evaluate the pleiotropic effect of the QTL identified for the specific traits with respect to other traits, as shown in the case of color traits in various organs (Figure 6). Legacy data also often contain evaluation of the overall traits judged as the totality of multiple individual specific traits (e.g. PT discussed in this paper, and also panicle structure and plant vigor), all of which are often evaluated by the breeders’ intuition and are important criteria for breeding selection. By identifying individual QTL that are involved in complex traits and examining their effects, we can evaluate the contribution of individual QTL to the complex traits. Therefore, combining our own acquired phenotypic data with legacy data will enable us to accelerate the progress of genetic research.

In the NARO Genebank, phenotypic data are provided not only for rice, but also for a variety of plant species, including wheat (*Triticum aestivum*), potato (*Solanum tuberosum*), grasses, fruit trees, and vegetables. In addition to the NARO Genebank data, there are reports investigating yield traits such as fertility, germination characteristics (Secretariat of Agriculture, Forestry and Fisheries Research Council., 1970), and eating quality characteristics of Japanese rice landraces (Sasahara et al., 2017). While these reports are on Japanese varieties, the IRRI SNP-SEEK phenotypic database (Mansueto et al., 2017) has collected phenotypic data from many countries for about two-thirds of the approximately 3,000 rice lines for which genomic data are available, and other trait data have been accumulated in various locations throughout the world. In the near future, databases linking genomic information with phenotypic data are expected to be released worldwide, and the research approach proposed here will be more effective. To accelerate genomic breeding, we propose the utilization of legacy data accumulated by our predecessors.

## Materials and methods

### Plant material and genotyping

We used two Japanese *japonica* rice (*Oryza sativa*) panels comprising 198 (for L-GWAS) and 172 (for M-GWAS) varieties, which were collected from various places in Japan (Supplemental Tables 1 and 2). DNA preparation and genotyping were conducted as previously described (Yano et al., 2016, 2019). After removing nucleotide variations with missing rates ≥0.1 and minor allele frequency <0.05, 179,700 SNPs and 26,147 insertions or deletions (INDELs) were identified in the 198 set, and 215,698 SNPs and 29,487 INDELs were found in the 172 set.

SnpEff software version 4.3 T (Cingolani et al., 2012) was used to predict the effect of genomic variants on gene function. The general feature format version 3 (gff3) from the Rice Genome Annotation Project (Ouyang et al., 2007; http://rice.plantbiology.msu.edu/) was used to provide information on gene position and coding sequences.

### Population genetic analyses and GWASs

The population structure of the 198 and 172 varieties was estimated using PCA performed using the R package “SNPRelate” version 4.2 (Zheng et al., 2012). For GWASs, we used a linear mixed model (LMM). For quantitative traits, GWASs were performed using the function *GWAS* in the R package “rrBLUP” version 4.3 with default parameter settings (Endelman, 2011). For binary traits, GWASs were performed using the function *association.test* in the R package “gaston” version 1.5.7 with default parameter settings (Perdry and Dandine-Roulland, 2018). In both GWASs, no fixed effects such as principal components were included. The genome-wide significant thresholds were determined using SimpleM which addresses the dependency among markers by calculating the number of effective markers (*M*_eff_) (Gao et al., 2008). In this study, 0.1/*M*_eff_ and 0.2/*M*_eff_ were used as the genome-wide significant thresholds. For comparisons of the results from L- and M-GWASs, an L-GWAS was performed including the polymorphism with the highest signal in the M-GWAS as a fixed effect to see if the peak disappeared (Method 1). If a peak detected in the L-GWAS is the same as a peak in the M-GWAS, the peak in the L-GWAS disappears when marker genotype data of the peak in the M-GWAS are included as a fixed effect in the GWAS model because explainability of the peak in the L-GWAS is removed by the fixed effect (Segura et al., 2012). In addition, correlations of −log_10_(*P*) of SNPs within the peak region were calculated (Method 2). When comparing L- and M-GWASs, only SNPs that were present in both L- and M-GWASs were extracted and analyzed for correlation. The effect size of each SNP was calculated using an in-house script that is a modification of the function *GWAS* in the R package “rrBLUP” version 4.3. To enable direct comparison of SNP effects between the traits, standardized phenotypic values were used for the calculation (mean = 0, SE = 1, calculated using Microsoft Excel). Then, the absolute values of the SNP effects were provided for the comparison.

### Phenotypic data

Phenotypic data were downloaded from the NARO Genebank (accessed August 5, 2022; https://www.gene.affrc.go.jp/distribution-plant_en.php). Original phenotypic data were scored according to the in-house manual (available in the NARO Genebank website), with five replications for measurement traits and observing the entire survey area for the observational traits. In the NARO Genebank, there are phenotypic data measured at 12 locations (Hokkaido, Aomori, Akita, Miyagi, Niigata, Fukui, Ibaraki, Hyogo, Hiroshima, Fukuoka, Okinawa, and Taiwan) from 1965 to 2020. The numerical data were used without modification. The Heading Date was converted to numerical data as days after July 1. Ordinal data, such as color traits, were converted to numerical data according to the scale in the NARO Genebank (Table 1). Basically, when phenotypic data existed for multiple years or at multiple locations, the phenotypic values were adjusted considering the location-by-year effect using a LMM. The LMM was implemented in the *lmer* function of the R package “lme4” version 1.1-31 (Bates et al., 2015). For Heading Date, two datasets were prepared (Heading Date at Niigata [HDN] and Integrated Heading Date recorded at all different locations [IHD]). In this study, 33 traits with sufficient data accumulated for GWASs were used. We produced a histogram of each trait and, based on observation of the distribution of data in the histograms, we designated 13 and 20 traits as binary and quantitative traits, respectively (Supplemental Figure 3). The trait correlation matrix was calculated using a Microsoft Excel analysis tool.

WC and Heading Date used for M-GWAS were surveyed at Togo Field, Field Science Center, Nagoya University, in 2014 and 2015, respectively. Heading Date is the number of days from planting to heading dates. The survey of BW was conducted at the experimental field of Fukui Prefectural University in 2014. These phenotypic data used for M-GWAS are shown in Supplemental Table 2. For the M-GWAS on Panicle Number (Supplemental Figure 11), the original data reported in Chigira et al. (2020) were provided by Prof. Taiichiro Ookawa (Tokyo University of Agriculture and Technology).

### Statistical analyses

Multiple comparison tests were performed using the *TukeyHSD* function and *anova* function in R.

### Accession numbers

Sequence data from this article can be found in the RGAP data libraries under the following accession numbers: *EPFL8/RAE2/GAD1, LOC_Os08g37890; waxy, LOC_Os06g04200; qSH1, LOC_Os01g62920; HESO1, LOC_Os01g62780; Hd1, LOC_Os06g16370; Hd2, LOC_Os07g49460; OsC1, LOC_Os06g10350; OsDFR, LOC_Os01g44260; NAL1, LOC_Os04g52479*.

## Supplementary Material

kiad018_Supplementary_Data

## Supplemental data

The following materials are available in the online version of this article.

**Supplemental Figure S1**. Genetic population structure of Japanese rice varieties used for M-GWAS.

**Supplemental Figure S2**. Pearson correlation coefficients between all 33 traits of legacy data from NARO Genebank.

**Supplemental Figure S3**. Histogram of all 33 traits of legacy data and 3 traits that we measured.

**Supplemental Figure S4**. Manhattan plots for L-GWAS of 18 traits.

**Supplemental Figure S5**. Q–Q-plots of L-GWAS of original data of all 33 traits.

**Supplemental Figure S6**. Q–Q plots of M-GWAS of 3 traits.

**Supplemental Figure S7**. GWAS for Integrated Heading Date (IHD).

**Supplemental Figure S8**. Method 2: Identity test for Seed width.

**Supplemental Figure S9**. Comparison between L- and M-GWASs for amount of White Core.

**Supplemental Figure S10**. Observation of ApC of 5 varieties with discrepancies between legacy data and genetic prediction of *OsC1*.

**Supplemental Figure S11**. Comparison between L-GWAS (Figure 7B) and M-GWAS of PN reported by Chigira et al. (2020).

**Supplemental Table S1**. List of the 198 varieties used for L-GWAS in this study.

**Supplemental Table S2**. List of the 172 varieties and phenotypic data used for M-GWAS in this study.
