# Supplementary material for: Effective use of legacy data in a genome-wide association studies improves the credibility of quantitative trait loci detection in rice
Source: Plant Physiol. 2023 Jan 18;191(3):1561–73. doi: 10.1093/plphys/kiad018 (PMC10022637; doi:10.1093/plphys/kiad018)
Supplement: kiad018_Supplementary_Data [file kiad018_supplementary_data.zip › Supplemental Figure.pdf]

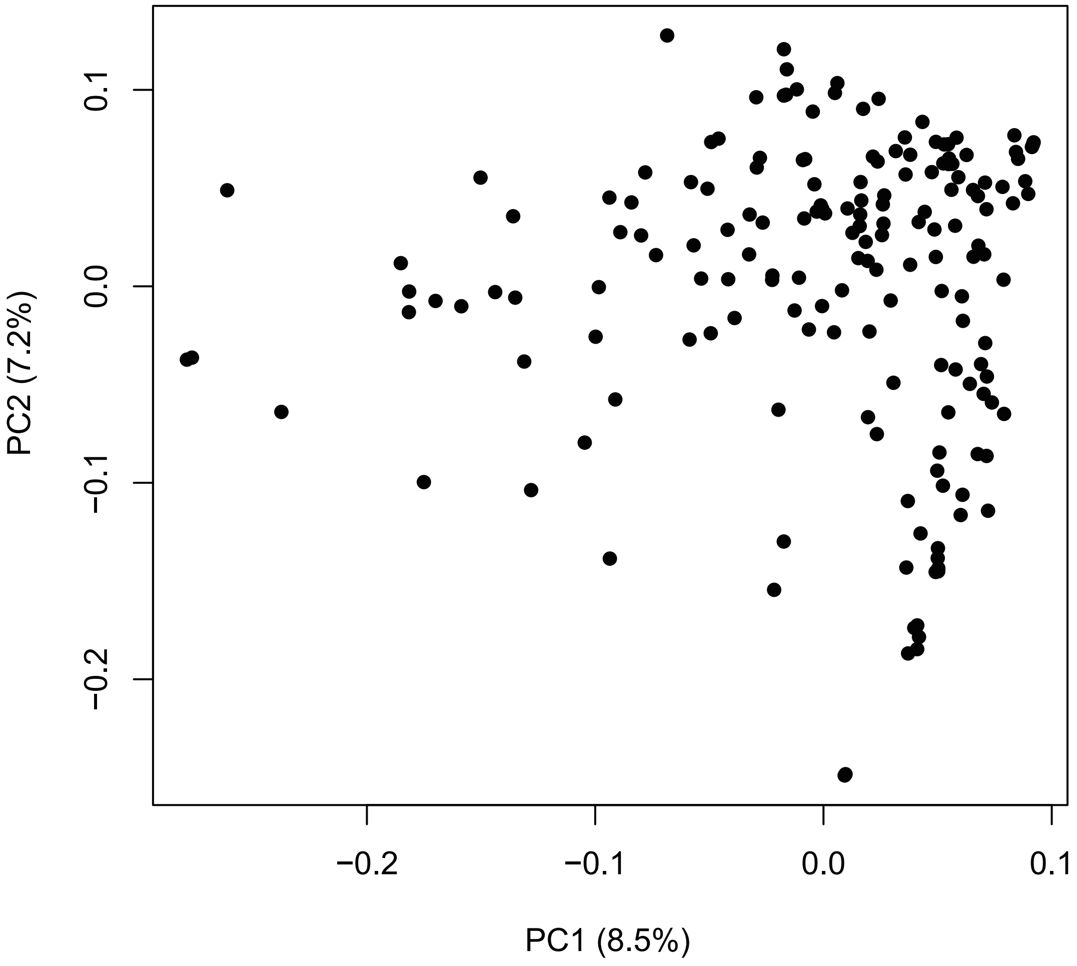

Supplemental Figure S1. Genetic population structure of Japanese rice varieties used for M-GWAS. PCA for the 172 Japanese rice varieties based on whole-genome data. PC1 and PC2 indicate the score of principal components 1 and 2, respectively. Values in parentheses indicate percentage of variance in the data explained by each principal component.

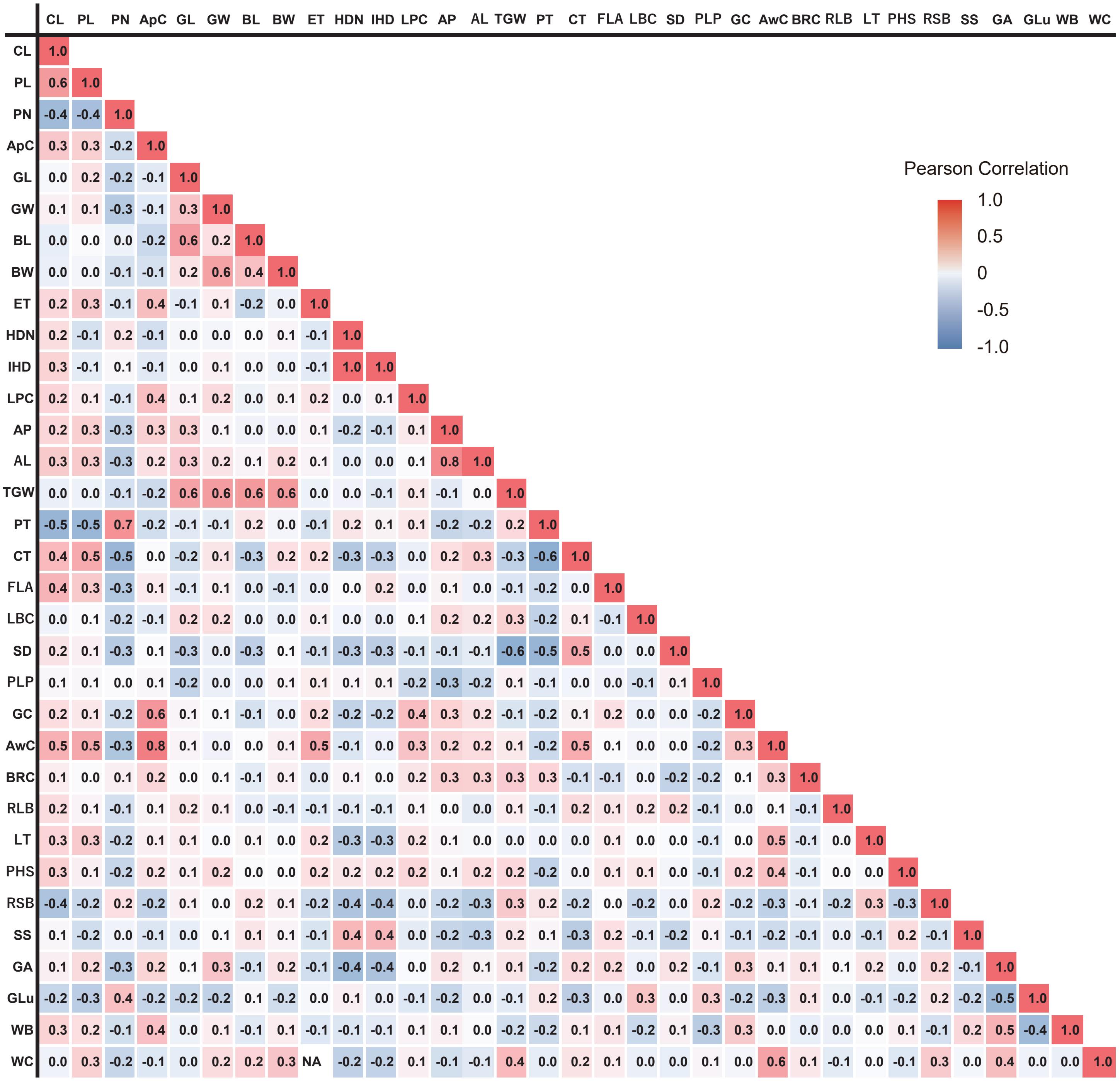

Supplemental Figure S2. Pearson correlation coefficients between all 33 traits of legacy data from NARO Genebank. Blue and red indicate negative and positive correlation, respectively. 33 traits are Culm Length (CL), Panicle Length (PL), Panicle Number (PN), Apiculus Color (ApC), Grain Length (GL), Grain Width (GW), Brown rice Length (BL), Brown rice Width (BW), Endosperm Type (ET), Heading Date at Niigata (HDN), Integrated Heading Date (IHD), Lemma and Palea Color (LPC), Awn Presence (AP), Awn Length (AL), 1,000 Grain Weight (TGW), Plant Type (PT), Culm Thickness (CT), Flag Leaf Angle (FLA), Leaf Blade Color (LBC), Spikelet Density (SD), Pubescence of Lemma and Palea (PLP), Glume Color (GC), Awn Color (AwC), Brown Rice Color (BRC), Resistance to Leaf Blast (RLB), Lodging Tolerance (LT), Pre-Harvest Sprouting (PHS), Resistance to Sheath Blight (RSB), Seed Shattering (SS), Grain Appearance (GA), Grain Luster (GLu), amount of White Belly (WB) and amount of White Core (WC).

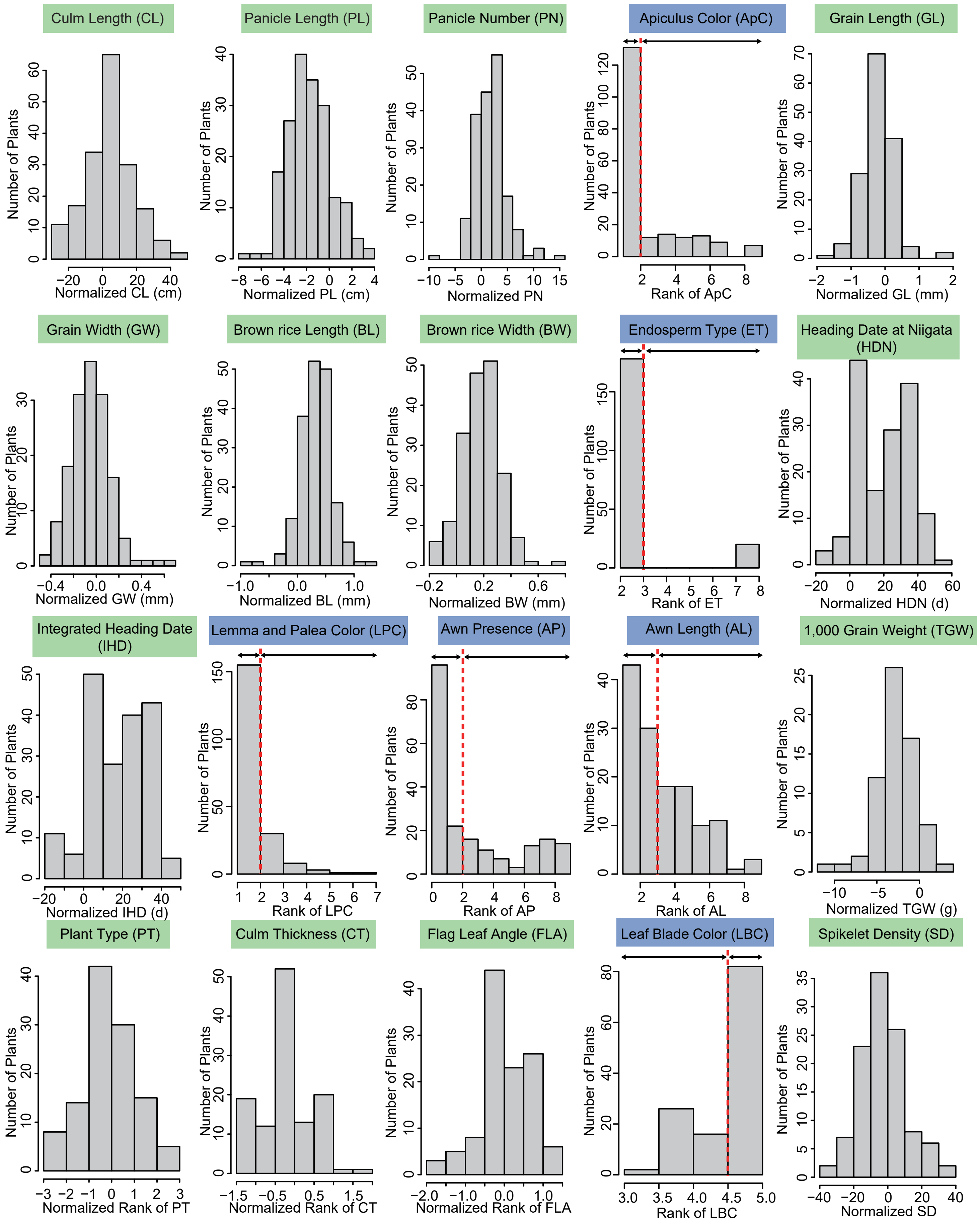

Culm Length (CL)
Panicle Length (PL)
Panicle Number (PN)
Apiculus Color (ApC)
Grain Length (GL)
Normalized CL (cm)
Normalized PL (cm)
Normalized PN
Rank of ApC
Normalized GL (mm)
Grain Width (GW)
Brown rice Length (BL)
Brown rice Width (BW)
Endosperm Type (ET)
Heading Date at Niigata (HDN)
Normalized GW (mm)
Normalized BL (mm)
Normalized BW (mm)
Rank of ET
Normalized HDN (d)
Integrated Heading Date (IHD)
Lemma and Palea Color (LPC)
Awn Presence (AP)
Awn Length (AL)
1,000 Grain Weight (TGW)
Normalized IHD (d)
Rank of LPC
Rank of AP
Rank of AL
Normalized TGW (g)
Plant Type (PT)
Culm Thickness (CT)
Flag Leaf Angle (FLA)
Leaf Blade Color (LBC)
Spikelet Density (SD)
Normalized Rank of PT
Normalized Rank of CT
Normalized Rank of FLA
Rank of LBC
Normalized SD
Number of Plants

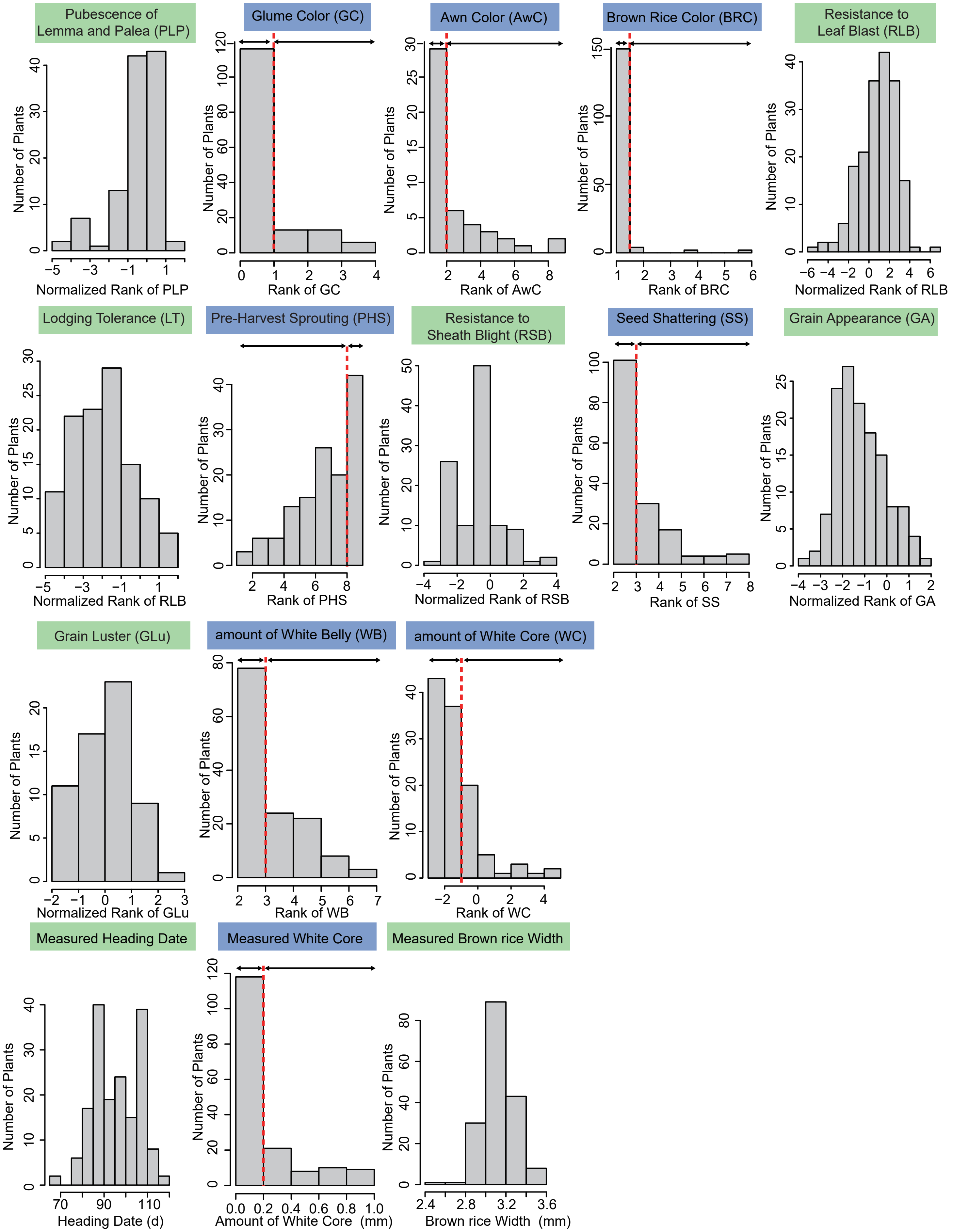

Supplemental Figure S3. Histogram of all 33 traits of legacy data and 3 traits that we measured. The abbreviation of the name of each trait and information on measurements are given in Table 1. The blue-highlighted 13 legacy traits (ApC, ET, LPC, AP, AL, LBC, GC, AwC, BRC, PHS, SS, WB, WC) and one measurement trait (White Core) were evaluated as binary traits and analyzed by binary GWAS because their variance was biased in one direction. The red line indicates the boundary where the trait was bisected. The green-highlighted traits were evaluated as quantitative traits and analyzed by quantitative GWAS.

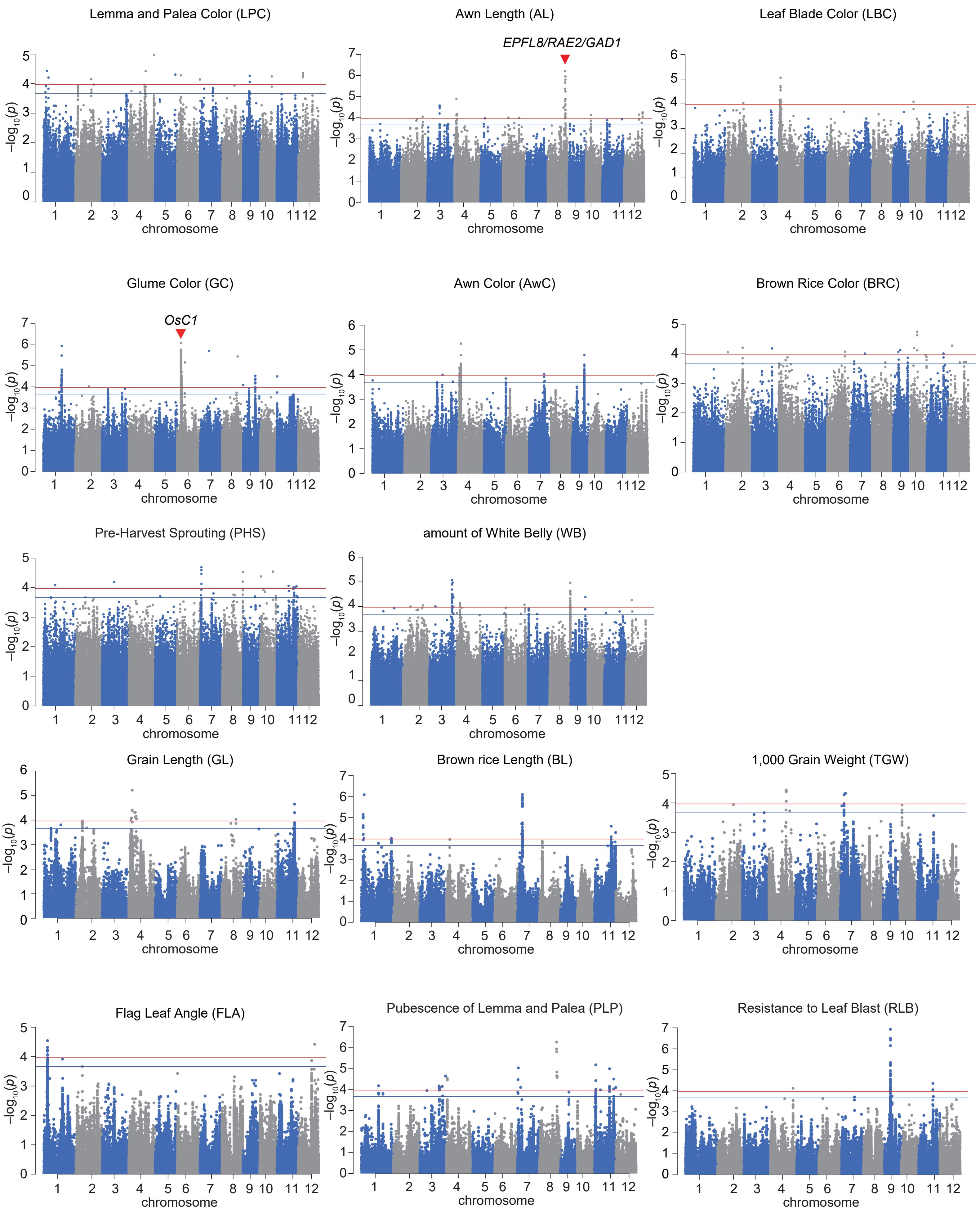
Lemma and Palea Color (LPC)
Awn Length (AL)
EPFL8/RAE2/GAD1
Leaf Blade Color (LBC)
Glume Color (GC)
OsC1
Awn Color (AwC)
Brown Rice Color (BRC)
Pre-Harvest Sprouting (PHS)
amount of White Belly (WB)
Grain Length (GL)
Brown rice Length (BL)
1,000 Grain Weight (TGW)
Flag Leaf Angle (FLA)
Pubescence of Lemma and Palea (PLP)
Resistance to Leaf Blast (RLB)
−log10(p)
chromosome

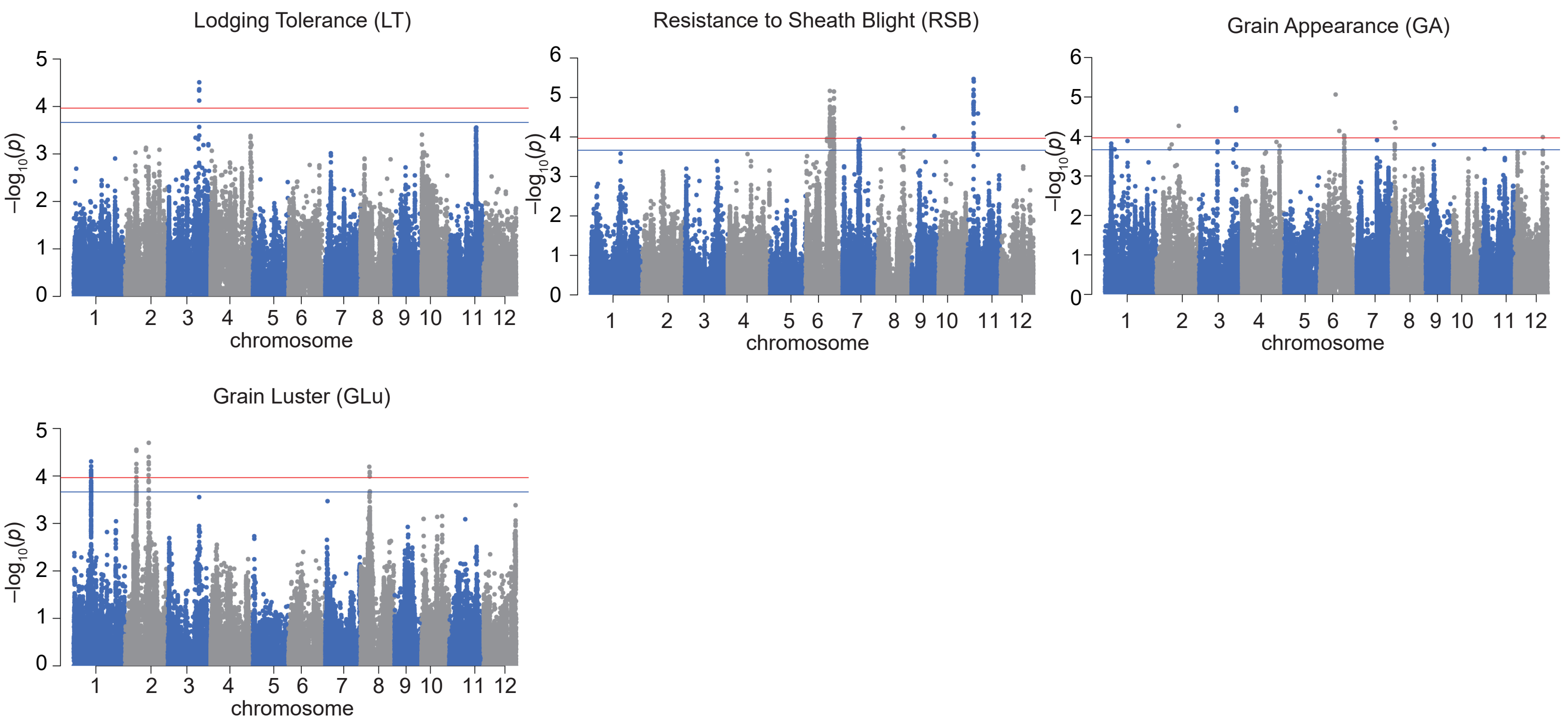

Supplemental Figure S4. Manhattan plots of L-GWAS of 18 traits. Manhattan plot of 18 traits not discussed elsewhere. The first eight traits were analyzed by binary GWAS, and the other 10 traits were by quantitative GWAS using linear scoring data. Arrowheads indicate the position of *EPFL8/RAE2/GAD1* in GWAS of AL and *OsC1* in GWAS of GC. Genome-wide significant threshold is indicated by horizontal lines (red: $0.1/M_{eff}$, blue: $0.2/M_{eff}$).

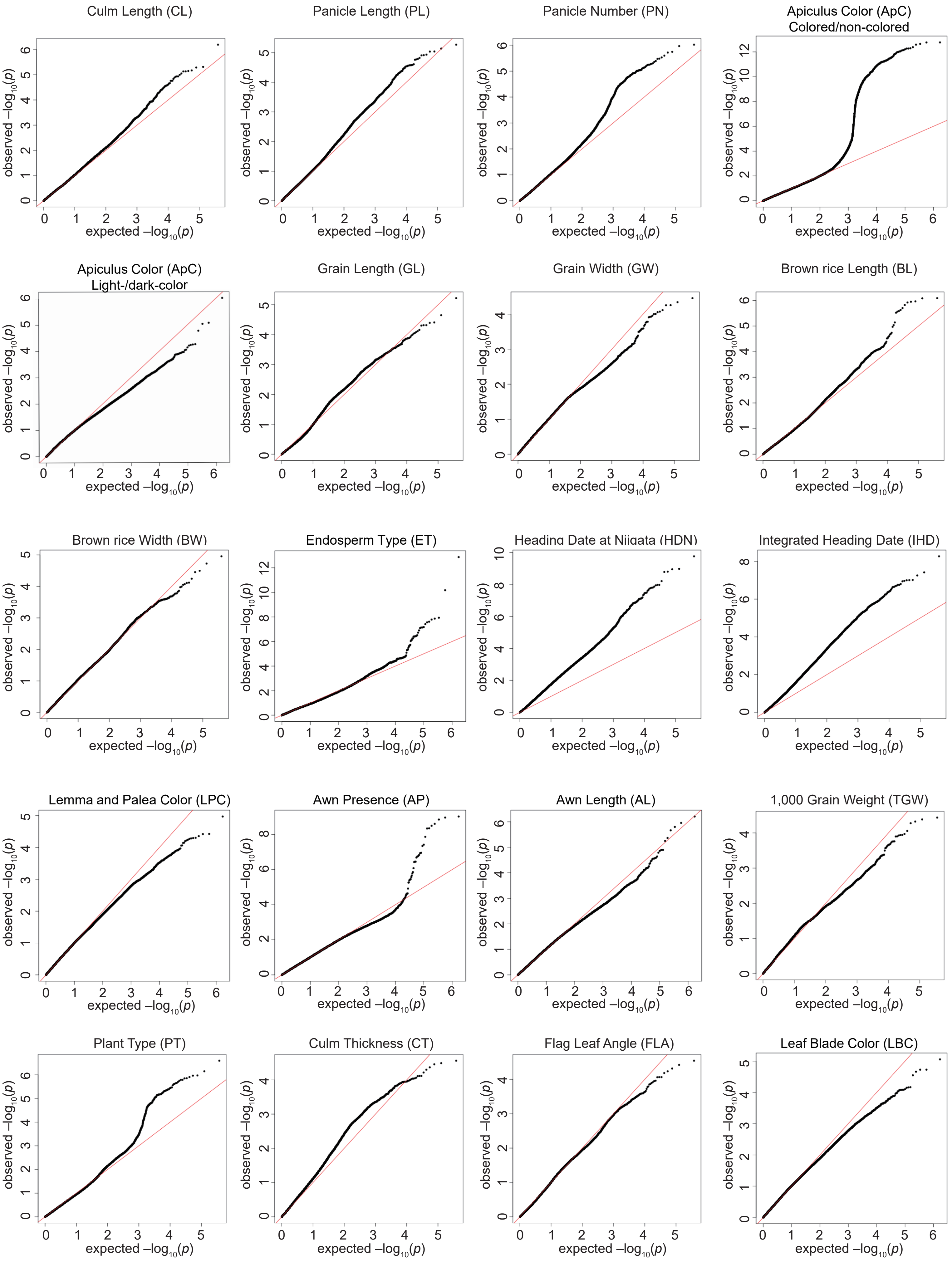
Culm Length (CL)
Panicle Length (PL)
Panicle Number (PN)
Apiculus Color (ApC)
Colored/non-colored
Apiculus Color (ApC)
Light-/dark-color
Grain Length (GL)
Grain Width (GW)
Brown rice Length (BL)
Brown rice Width (BW)
Endosperm Type (ET)
Heading Date at Niigata (HDN)
Integrated Heading Date (IHD)
Lemma and Palea Color (LPC)
Awn Presence (AP)
Awn Length (AL)
1,000 Grain Weight (TGW)
Plant Type (PT)
Culm Thickness (CT)
Flag Leaf Angle (FLA)
Leaf Blade Color (LBC)
observed $-\log_{10}(p)$
expected $-\log_{10}(p)$

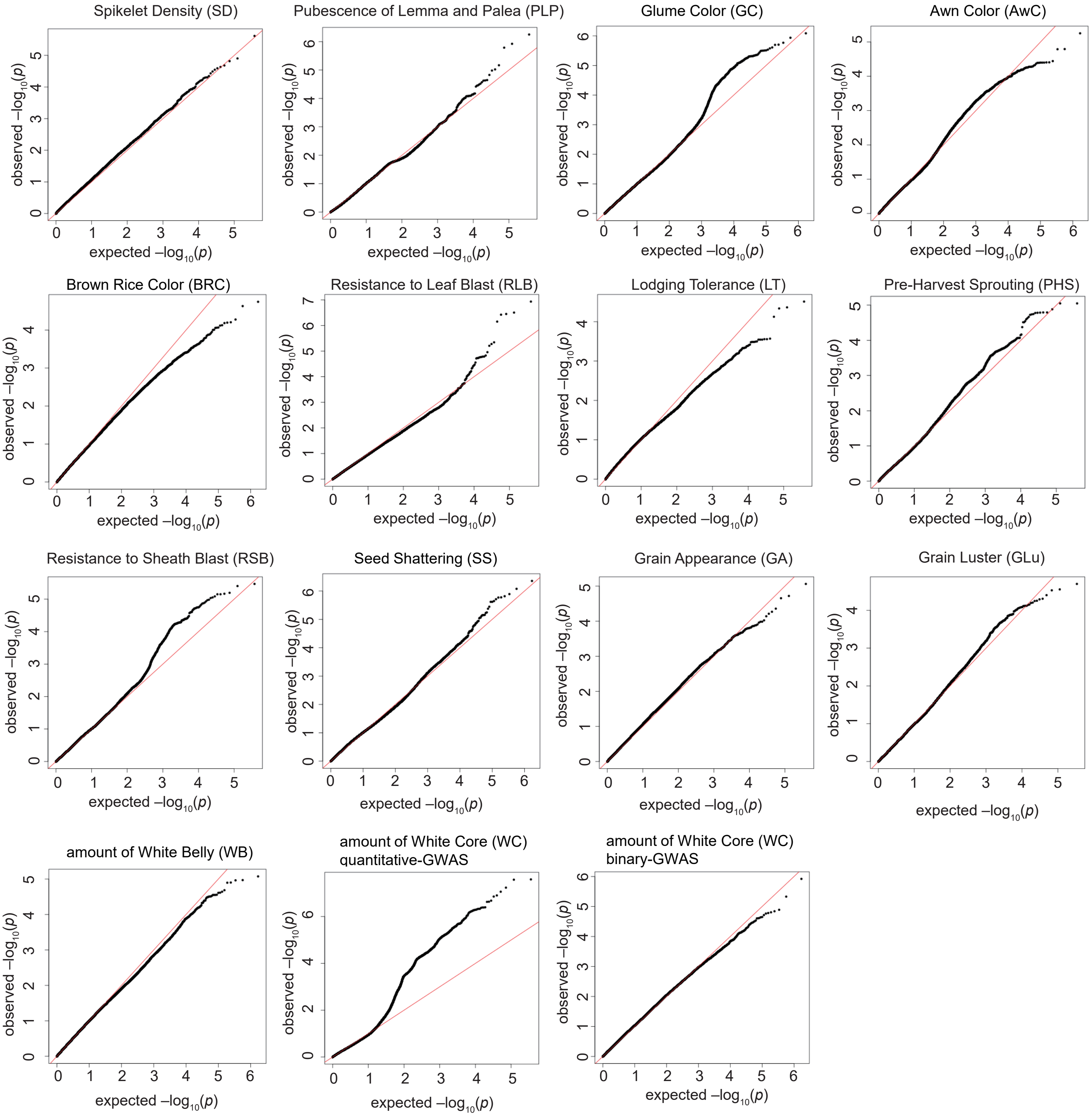

Supplemental Figure S5. QQ-plots of L-GWAS of original data of all 33 traits. The red line represents an ideal case where theoretical test statistic quantiles match the simulated test statistic quantiles.

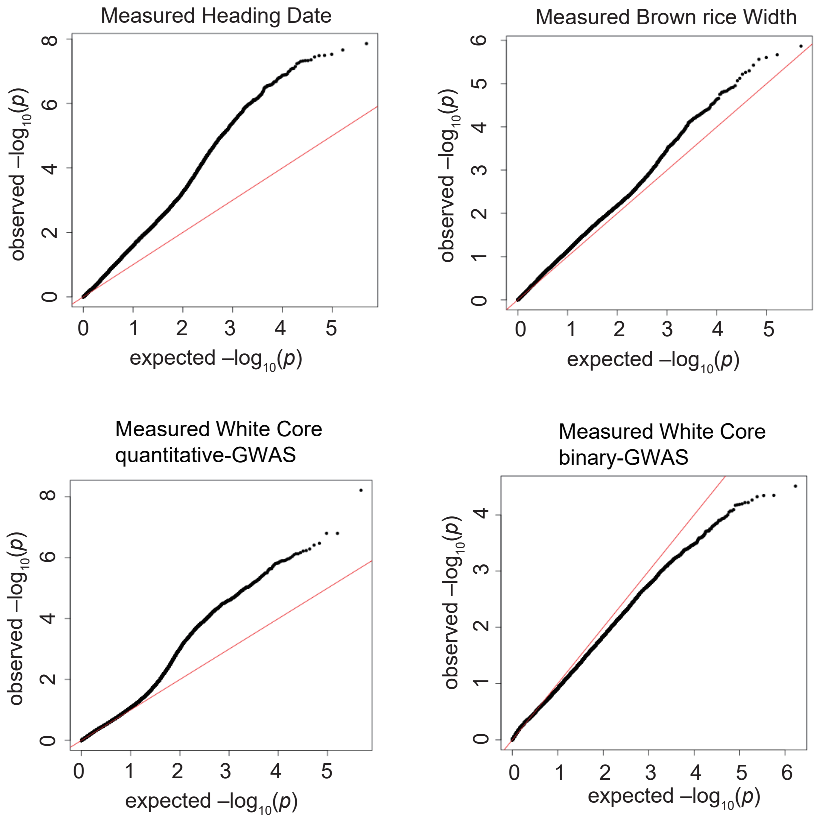

Supplemental Figure S6. QQ plots of M-GWAS of 3 traits. The red line represents an ideal case where theoretical test statistic quantiles match the simulated test statistic quantiles.

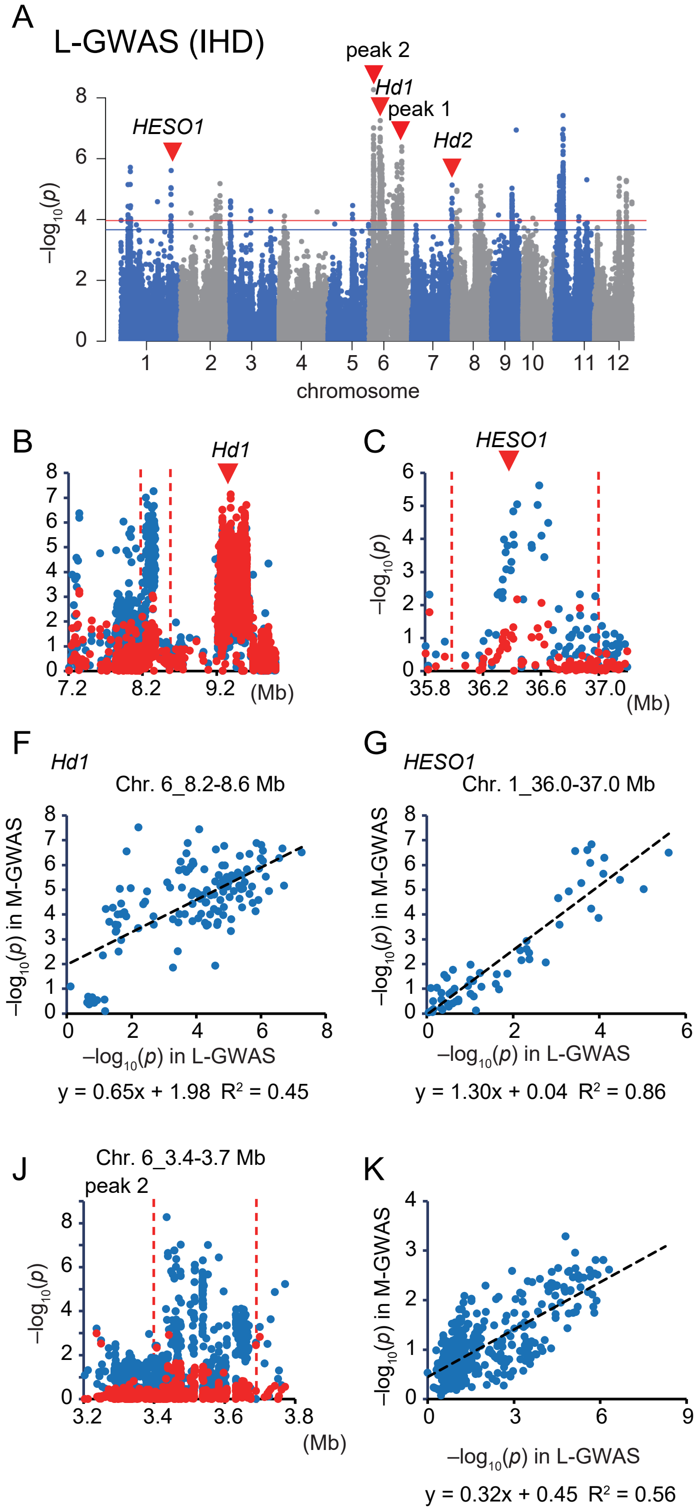

Supplemental Figure S7. GWAS for Integrated Heading Date (IHD). (A) Manhattan plot of IHD. Genome-wide significant threshold is indicated by horizontal lines (red: 0.1/ M_{eff}, blue: 0.2/ M_{eff}). (B-E, J) Local Manhattan plot of L-GWAS surrounding the peak region indicted by red arrowheads in panel (A). Plots showed the results of L-GWAS performed without (blue) or with (red) the polymorphism with the highest signal in M-GWAS as fixed effect. Red arrowheads indicate the position of heading genes, that is *Hd1* (B), *HESO1* (C), and *Hd2* (D), while there is no known heading gene in panel (E, J). Dashed lines indicate the candidate region for the peak. (F-I, K) Correlation of the $-\log_{10}(P)$ value of SNPs between L- and M-GWAS within the peak regions of *Hd1*, *HESO1*, *Hd2*, peak 1 and peak 2.

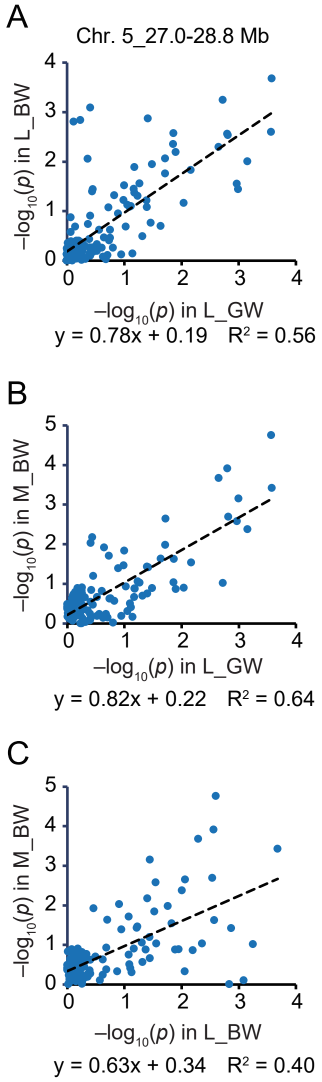

Supplemental Figure S8. Method 2: Identity test for Seed width. Correlation of the $-\log_{10}(P)$ value of SNPs between L_GW, L_BW and M_BW within the peak region indicted by red arrowheads in Figure 4, A-C.

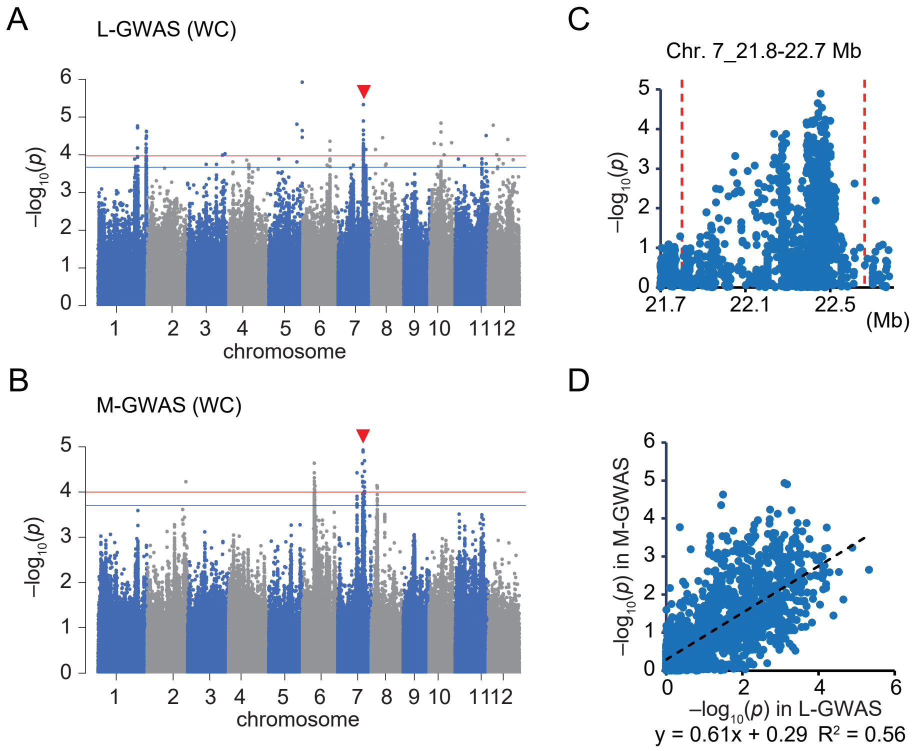

Supplemental Figure S9. Comparison between L- and M-GWAS for amount of White Core. (A, B) Manhattan plot of amount of White Core (WC) by using legacy data (A) and measured data (B). GWAS was performed using GWAS plantform for binary traits. Genome-wide significant threshold is indicated by horizontal lines (red: 0.1/ M_{eff}, blue: 0.2/ M_{eff}). (C) Local Manhattan plot of L-GWAS surrounding the peak regions indicted by red arrowheads in panel (A, B). (D) Correlation of the $-\log_{10}(P)$ value of SNPs between L- and M-GWAS within the peak region.

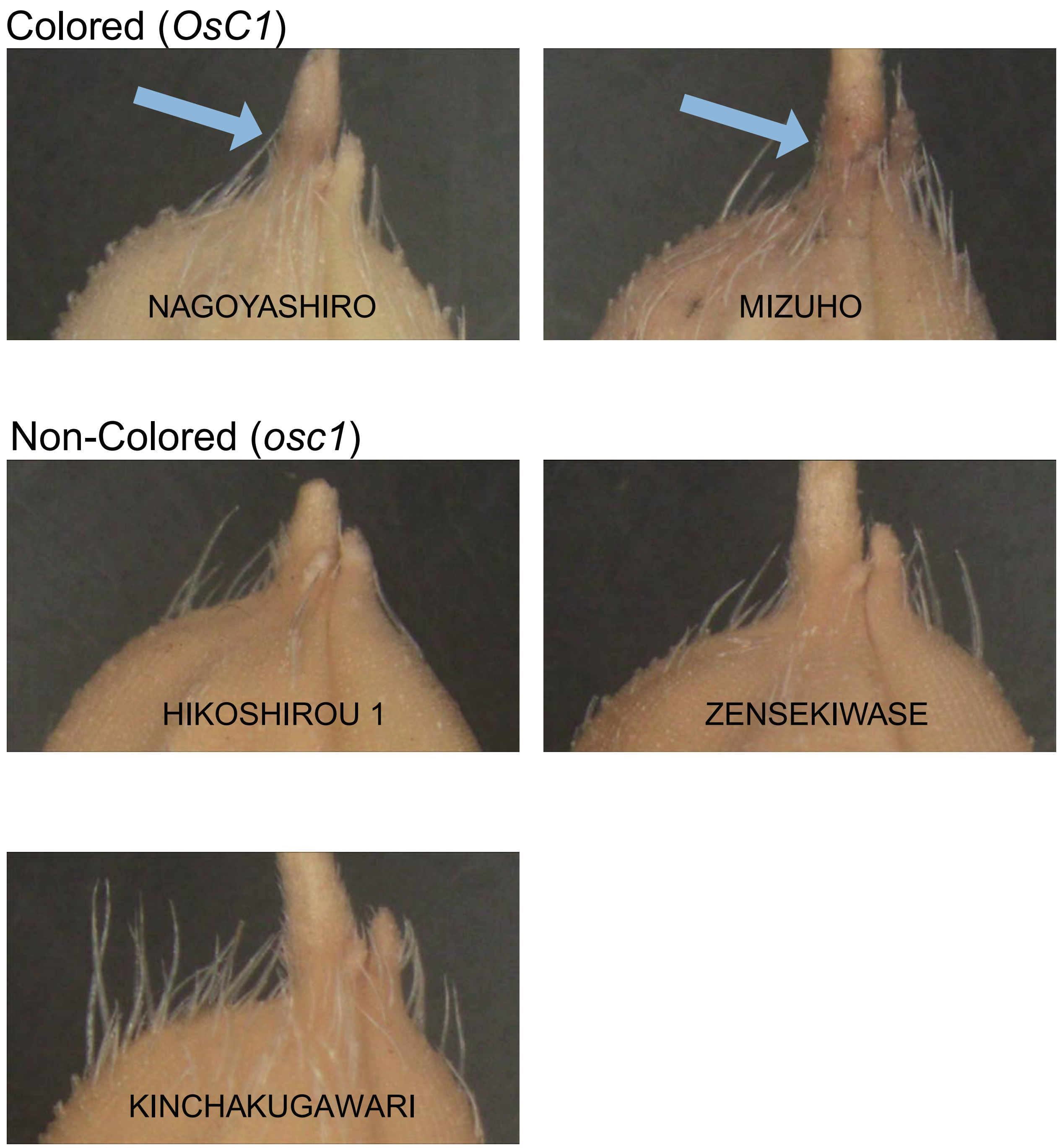

Supplemental Figure S10. Observation of ApC of five varieties with discrepancies between legacy data and genetic prediction of *OsC1*. NAGOYASHIRO and MIZUHO with the active *OsC1* haplotype show colored ApC, while HIKOSHIROU1, KINCHAKUGAWARI, and ZENSEKIWASE with the *osc1* haplotype show non-colored ApC, indicating that the trait predicted by *OsC1* is correct and the legacy data is incorrectly described.

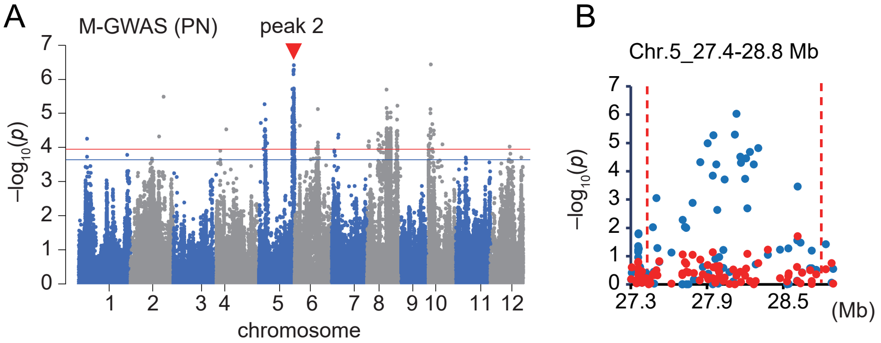

Supplemental Figure S11. Comparison between L-GWAS (Figure 7B) and M-GWAS of PN reported by Chigira et al. (2020). (A) Manhattan plot of M-GWAS using PN data of Chigira et al., (2020). Red arrowheads indicate a peak 2 observed in PT and PN of L-GWAS (Figure 7, A and B). Genome-wide significant threshold is indicated by horizontal lines (red: $0.1/M_{eff}$, blue: $0.2/M_{eff}$). (B) Local Manhattan plot of L-GWAS of PN surrounding peak 2. Plots showed the results of L-GWAS performed without (blue) or with (red) the polymorphism with the highest signal in M-GWAS as fixed effect, respectively.
